# Assessment of clinical benefit, cost and uptake of biosimilars versus reference biologics in immune-mediated inflammatory diseases in China

**DOI:** 10.3389/fpubh.2024.1476213

**Published:** 2024-12-04

**Authors:** Xin Du, Xingxian Luo, Qixiang Guo, Xiaomeng Jiang, Ziling Su, Weiting Zhou, Zhongjian Wang, Jiarun Li, Yue Yang, Yi Zhang

**Affiliations:** ^1^Vanke School of Public Health, Tsinghua University, Beijing, China; ^2^Department of Pharmacy, Peking University People's Hospital, Beijing, China; ^3^School of Pharmaceutical Sciences, Tsinghua University, Beijing, China; ^4^Key Laboratory of Innovative Drug Research and Evaluation, National Medical Products Administration, Beijing, China; ^5^School of Pharmaceutical Sciences, Sun Yat-sen University, Guangzhou, China; ^6^School of Pharmacy, Massachusetts College of Pharmacy and Health Sciences University, Boston, MA, United States; ^7^BCPMdata Pharma Technology (Chengdu) Co., Ltd, Chengdu, China

**Keywords:** immune-mediated inflammatory diseases, biosimilars, clinical benefit, cost, uptake

## Abstract

**Background:**

As China is one of the countries with the highest recorded cases of Immune-Mediated Inflammatory Diseases (IMIDs), these diseases have also emerged as a serious public health concern. Biosimilars, potentially lower-cost versions of biologics, may improve access to more affordable yet comparably effective treatments. Encouragingly, China launched its abbreviated biosimilar pathway in 2015, and since then, a large number of biosimilars have been approved. However, systematic studies on the therapeutic efficacy and economic impact of IMIDs biosimilars are lacking in China. This study aims to assess the clinical benefits (including efficacy/effectiveness, safety, and immunogenicity), cost and uptake of adalimumab biosimilars, tocilizumab biosimilars, and infliximab biosimilars compared with their reference biologics in patients with IMIDs in China.

**Methods:**

IMIDs biosimilars and their reference drugs approved in China between 2015 and 2024 were identified. Head-to-head randomized clinical trials (RCTs) and real-world cohort studies on adalimumab, tocilizumab and infliximab and their biosimilars for the treatment of IMIDs were assessed. PubMed, Embase, Cochrane Library, Clinicaltrials.gov, and Listed Drug Database of China National Medical Products Administration were searched for clinical trials and cohort studies on biosimilars for IMIDs from their inception to November 1, 2024. We evaluated the monthly treatment costs and quarterly uptakes of these biosimilars and their reference biologics in China. Besides, we simulated the impact of biosimilar substitution in different scenarios. Meta-analyses were performed using a random-effects model to evaluate the efficacy, safety, and immunogenicity of treatments, including pooled risk ratios (RR) for ACR20 for rheumatoid arthritis, ASAS20 for ankylosing spondylitis, and PASI for plaque psoriasis, treatment-emergent adverse events (TEAEs), serious adverse events (SAEs), anti-drug antibodies (ADAs), and neutralizing antibodies (Nabs), with 95% credible intervals (CrIs).

**Findings:**

A total of 12 RCTs involving 5,717 patients with IMIDs were analyzed, including 12 approved biosimilars of adalimumab, infliximab, and tocilizumab. The primary endpoints of adalimumab (7 RCTs with 3,174 patients; RR, 1.02; 95% CrI, 0.99–1.06, *p* = 0.33), infliximab (3 RCTs with 1,291 patients; RR, 1.02; 95% CrI, 0.94–1.11, *p* = 0.98), tocilizumab (2 RCTs with 1,252 patients; RR, 1.01, 95% CrI, 0.94–1.08) met equivalence with reference biologics. Additionally, there was no significant difference between biosimilars and their reference biologics in the secondary endpoints. Overall, biosimilars demonstrated comparable safety (TEAEs: RR, 0.99; 95% CrI, 0.95–1.02, *p* = 0.44) (SAEs: RR, 0.80; 95% CrI, 0.42–1.54, *p* = 0.50) and immunogenicity (ADA: RR, 1.00; 95% CrI, 0.95–1.04, *p* = 0.85) (Nabs: RR, 0.93; 95% CrI, 0.82–1.05, *p* = 0.25) profiles to reference biologics. These findings were consistent with the cohort studies. In 2024, IMIDs biosimilars are available at 63 to 82% of the price per unit of the reference drugs, with uptake rates of 16.5 to 72.1% in China. Patients with IMIDs using these biosimilars could save between $874 and $2,184 per month in treatment costs, equivalent to 1.8 to 7.0 times the *per capita* monthly disposable income in China in 2024. Simulation showed that with 100% biosimilar substitution, savings would increase to $22.98 M, $33.83 M, and $3.82 M for adalimumab, infliximab, and tocilizumab, respectively. This would enable treatment for an additional 6,700, 9,863, and 4,373 patients, respectively.

**Interpretation:**

Our study revealed that IMID biosimilars in China provide clinical benefits comparable to their reference biologics evidenced by high-quality RCTs and cohort studies with offer significant cost savings in China. Encouraging China’s national volume-based procurement and multi-stakeholder collaboration may help accelerate the substitution of IMIDs biosimilars.

## 1 Introduction

Immune-mediated inflammatory diseases (IMIDs) are characterized by excessive and uncontrolled inflammation and are highly prevalent across a group of conditions. These conditions are often accompanied by severe cardiovascular, metabolic disorders, cognitive impairment, and other complications, which severely impact the quality of life of patients (1). Common IMIDs include rheumatoid arthritis (RA), ankylosing spondylitis (AS), plaque psoriasis, Crohn’s disease and uveitis (2). Over the past two decades, there have been landmark achievements in the treatment of IMIDs, especially with the development of monoclonal antibody-based biologics, which have significantly improved patient symptoms (3). For example, tumor necrosis factor-*α* inhibitors, such as infliximab, adalimumab, and tocilizumab are biologic disease-modifying antirheumatic drugs used worldwide to treat IMIDs. The latest annual sales figures forecast that adalimumab ranks fourth globally, with expected sales of $13.6 billion (4). However, it should be noted that the high cost of IMIDs drugs remains a serious concern for patients, prescribers, and payors even in high-income countries (5).

Biosimilars, drugs that are highly similar in structure, efficacy and safety to the approved reference drugs, are often significantly less expensive than the reference drugs, promising to alleviate the burden on patients (6). Hence, to address the high expense of novel biologics, abbreviated approval pathway for biosimilars were established in the EU (2006), Japan (2009), and the US (2010), to improve affordability for patients (7). As of November 1, 2024, the European Medicines Agency (EMA) (8), the US Food and Drug Administration (FDA) (9), and the Japanese Pharmaceuticals and Medical Devices Agency (PMDA) (10), have approved more than 97, 50, and 41 biosimilars, respectively. Among them, the number of biosimilar approvals for adalimumab, infliximab and tocilizumab for the treatment of IMIDs reaches up to 37 (Supplementary Table S1). Previous studies have reported that biosimilars offer potentially cost-effective treatment options for autoimmune diseases (11, 12). However, as with other biosimilars, the use of biosimilars for IMIDs remains confronted with many challenges, including uncertainty about clinical benefits, patent and data exclusivity, substitution and interchangeability policies, and healthcare payments (13). These issues have contributed to the suboptimal use of biosimilars (14, 15).

In China, the prevalence of IMIDs is among the highest in the world, making these diseases a critical public health concern (16). The prevalence of RA in China was reported to be 0.42%, with a total affected population of about 5 million, making it the second leading cause of disability in in the country (17). The annual total cost of RA in China was about ¥12.67 billion RMB, among which direct medical costs accounting for 33.3%, with direct non-medical costs account for 29.8% and indirect costs account for 37.0% (18). In 2015, the China Food and Drug Administration (now known as the National Medical Products Administration, NMPA) established a streamlined biosimilar approval pathway and issued a series of policies in the field of biosimilars aimed at clarifying the definition and regulatory principles of biosimilars while encouraging their development (13, 19, 20). More importantly, the NMPA has developed specific guidelines for adalimumab biosimilars and infliximab biosimilars. These incentives have indeed led to the development of local biosimilars. As of November 1, 2024, China has approved 15 biosimilars for the treatment of IMIDs, with another 25 biosimilars in the pipeline (Supplementary Tables S1–S3). However, evidence of the clinical benefits, cost, and uptake of these biosimilars for the treatment of IMIDs is rarely reported in China.

To address the issues mentioned above, this study aims to compare the evidence for IMIDs biosimilars with their reference drugs by including RCTs and real-world cohort studies assessing in efficacy/effectiveness, safety, and immunogenicity. Furthermore, it evaluates the cost and uptake of these biosimilars versus their reference drugs after the biosimilars’ entry into the market. This evidence may facilitate a better understanding of the value of biosimilars and promote their adoption and utilization in China.

## 2 Methods

### 2.1 Data sources

Head-to-head RCTs and cohort studies on biosimilars of adalimumab, infliximab and tocilizumab and their reference biologics for IMIDs were assessed. PubMed, Embase, Cochrane library, Clinicaltrials.gov and Listed Drug Database of Center for Drug Evaluation, NMPA (21) were searched from their inception to November 1, 2024. Price and uptake data from the launch date to July 1, 2024 were retrieved from the Pharnexcloud database (22).

### 2.2 Data extraction

#### 2.2.1 Identification of RCTs and cohort studies

This study included RCTs and cohort studies that involved patients with IMIDs. The intervention groups consisted of patients treated with any biosimilars of adalimumab, infliximab, or tocilizumab, while control groups consisted of patients who received respective reference drugs. No restrictions were applied to the dosage, treatment, regimen, or patient number. Non-comparative studies (e.g., reviews, expert commentary, editorials, and clinical guidelines) were excluded. A detailed description of the eligibility and exclusion criteria can be found in Appendix 1. Two investigators (X.D., X.L.) extracted the data of IMIDs biosimilars from RCTs and cohort studies, including patient numbers, whether the study was sponsored by a manufacturer, study design, duration of study, therapy lines, endpoints for efficacy/effectiveness, safety, and immunogenicity. The primary endpoints of efficacy included American College of Rheumatology 20% improvement criteria (ACR20), Psoriasis Area and Severity Index (PASI), and Assessment of Spondylo Arthritis international Society 20% improvement criteria (ASAS20). The safety outcomes included Treatment Emergent Adverse Event (TEAE), Serious Adverse Event (SAE), Drug-related TEAE hypersensitivity, drug interruption and drug discontinuations. In addition, immunogenicity outcomes included the incidence of neutralizing antibodies (Nabs) and anti-drug antibodies (ADA).

#### 2.2.2 Price, cost and uptake data extraction

##### 2.2.2.1 Price and cost

China has a three-tier healthcare delivery system, with healthcare institutions and providers operating at different levels: county, township, and village levels in rural areas, and at the municipal, district, and community levels in urban areas (23, 24). We extracted the winning bid price from each province to calculate the annual weighted average price of biosimilars per milligram. Meanwhile, we adjusted the price of biosimilars to reflect 2024 values, accounting for inflation. Finally, we converted these values into US dollars based on the 2024 exchange rate between the Chinese Yuan (RMB) and the US Dollar (USD) in 2024, which was 1 USD to 7.1 RMB (25).

In addition, we calculated the monthly treatment costs for both reference biologics and biosimilars individually, using information from the drug labels. The annual treatment cost was determined by multiplying the dose administered to a patient over 1 year (52 weeks) by the unit price. Subsequently, we calculated the average monthly treatment cost over 12 months.

##### 2.2.2.2 Uptake

In China, healthcare institutions are categorized into primary healthcare institutions, secondary comprehensive hospitals and tertiary comprehensive hospitals. Normally, higher-level institutions provide more sophisticated treatments. In this study, we utilized the national hospital sales volume data from the Pharnexcloud database module, which covered the sales volumes of cancer biosimilars in secondary and tertiary hospitals nationwide, as there was no public database available for drug sales volumes (26). We separately extracted the sales volume data of biosimilars and reference drugs in China for each quarter. The uptake of biosimilar was defined as the ratio of biosimilar sales volume to the total sales volume (combining sales volumes of both biosimilars and reference drugs). The dataset spans from the introduction of biosimilars to the market until October 1, 2023. A detailed description of price, cost and uptake of biosimilars and reference drugs in China can be found in Appendix 2.

##### 2.2.2.3 Simulation

To better evaluate the effect of biosimilar substitution, we designed two scenarios for simulation, including biosimilar substitution in 2023 and 100% of biosimilar substitution. Since the data for 2024 was still incomplete, the simulation took the annual treatment cost, sales volume and revenue of the reference biologics and biosimilars in 2023 as the baseline. Given the prices of different biosimilars vary, the annual treatment cost of biosimilars is weighted according to their market share. The specific calculations can be found in Supplementary Table S12.

### 2.3 Assessment of risk of bias

Two investigators (X.D., X.L.) assessed the risk of bias in the RCTs and cohort studies in accordance with the Cochrane Collaboration’s tool (CCT) and Newcastle-Ottawa Risk of Bias Assessment Tool (NOS). The assessment of CCT included selection bias (sequence generation and allocation concealment), performance bias (blinding of participants, personnel, and outcome assessors), attrition bias (incomplete outcome data), reporting bias (selective outcome reporting), and other potential biases (27). There are three main dimensions involved in NOS, including selection, comparability and outcome (28).

### 2.4 Statistical analysis

Medians (IQRs) were used for continuous variables. Counts and percentages were used for categorical variables. Similar to the previous study, we calculated the median weighted average price (WAP) separately when there were multiple biosimilars approved (13). For biosimilars or reference drugs with different dose strengths, price were standardized to price per milligram (eg, adalimumab is available in 40 mg per vial or 20 mg per vial in China) to facilitate comparison. The uptake rate of biosimilars was defined as the ratio of quarterly (3-month) sales volume of the biosimilar to combined quarterly sales volume of the biosimilar and its reference drugs as the sales volume was obtained quarterly.

We pooled relative estimates of adalimumab biosimilars, infliximab biosimilars and tocilizumab biosimilars compared to their respective reference drugs, considering the significant differences in indications and mechanisms of the tested drugs. This study also analyzed subgroups of these biosimilars for primary and secondary endpoints (efficacy/effectiveness, safety and immunogenicity) and indications. Considering the possible heterogeneity of different biosimilars in terms of design, patient population, and efficacy endpoints, restricted maximum likelihood random-effects meta-analysis was used for pooling efficacy, safety and immunogenicity outcomes of IMIDs biosimilars, following methodologies from a previous study (24).

Statistical analyses were performed and graphical representations were generated using IBM SPSS, (version 20 IBM Corp) and R (version 4.1.0 R Project for Statistical Computing). The R packages used in the analysis included meta (version 5.2.0), forestplot (version 1.10.1), and ggplot2 (version 3.4.0). Two-sided tests were conducted with a significance threshold of *p* < 0.05.

## 3 Results

### 3.1 RCT and cohort study characteristics

Table 1 summarizes the characteristics of the 12 RCTs, involving 5,717 patients with IMIDs, with a median (IQR) sample size of 482 (370 to 601) patients. Of those, 9 RCTs were published in journals (26, 29–36), and 3 were reported in NMPA reviews (37–39). All identified studies were funded by various sponsors, encompassing 12 biosimilars marketed across 3 disease settings in China: plaque psoriasis (PP), ankylosing spondylitis (AS) and rheumatoid arthritis (RA). Among the 12 RCTs, 7 studied adalimumab biosimilars (26, 29–33, 37), 3 studied infliximab biosimilars (34, 35, 38), and 2 studied tocilizumab biosimilars (36, 39).

**Table 1:** Characteristics of the included randomised controlled trial.

Source	Indication	Biosimilar drug	Reference drug	Study design	Patients, No.	Females, No. /males, No.	Primary endpoint	Study duration (month)
Yu et al., 2022	PP	Adalimumab-SCT630	Adalimumab	Equivalence	367	70/297	PASI (16w)	21.93
TopAlliance, 2022	RA	Adalimumab-UBP1211	Adalimumab	Equivalence	526	NA	ACR20 (24w)	NA
Li et al., 2022	AS	Adalimumab-TQZ2301	Adalimumab	Equivalence	380	51/329	ASAS20 (24w)	13.17
Xu et al., 2020	AS	Adalimumab-IBI303	Adalimumab	Equivalence	438	76/362	ASAS20 (25w)	19.87
Su et al., 2021	AS	Adalimumab-HS016	Adalimumab	Equivalence	648	85/563	ASAS20 (26w)	5.13
Cai et al., 2020	PP	Adalimumab-HLX03	Adalimumab	Equivalence	261	71/190	PASI (16w)	NA
Tu et al., 2019	AS	Adalimumab-BAT1406	Adalimumab	Equivalence	554	75/479	ASAS20 (12w)	7.07
Hisun et al., 2021	PP	Infliximab-HS626	Infliximab	Equivalence	337	NA	PASI (12w)	NA
Liu et al., 2022	RA	Infliximab-GB242	Infliximab	Equivalence	570	477/89	ACR20 (30w)	29.40
Ye et al., 2021	RA	Infliximab-CMAB008	Infliximab	Noninferiority	384	325/59	ACR20(31w)	16.57
Lizhu et al., 2023	RA	Tocilizumab-LZM008	Tocilizumab	Equivalence	640	NA	ACR20 (24w)	NA
Leng et al., 2023	RA	Tocilizumab-BAT1806	Tocilizumab	Equivalence	612	NA	ACR20 (24w)	31.50

NA, not applicable. RA, Rheumatoid arthritis. AS, Ankylosing spondylitis. PP, Plaque psoriasis. PASI, psoriasis area and severity index. ACR20, American College of Rheumatology 20% Response Criteria.

The majority of the RCTs were designed as equivalence designs, while only Infliximab-CMAB008 was prespecified as a noninferiority design. The median (IQR) proportion of females was 14% (13 to 22%) for AS, 23% (19 to 27%) for PP, and 84.5% (84 to 85%) for RA. The median (IQR) study duration for assessing the primary efficacy endpoints was 10.12 (5.62–18.20) months for AS, 21.93 months for PP, and 29.4 (16.57–31.50) months for RA, respectively. The risk of bias assessment in RCTs, as detailed in Supplementary Table S5, indicated that 8 RCTs (66.7%) were of low risk and 4 were at uncertain risk.

Four cohort studies with 3,918 comparing biosimilars with their reference drugs were found (40–43). All cohort studies were conducted out of China and were rated as low risk (Supplementary Tables S4, S6) summarize the characteristics of the 4 cohort studies with a total of 3,918 patients. Of the 4 cohort studies, 2 (50.0%) focus on a adalimumab biosimilar (40, 41), 2 (50.0%) were of infliximab biosimilars (42, 43).

### 3.2 Clinical benefits

#### 3.2.1 Adalimumab biosimilars vs. adalimumab

Seven RCTs compared adalimumab biosimilars with the originator (26, 29–33, 37), four studies including patients with AS (30–33), two studies including patients with PP (26, 29), and one study including patients with RA (37). The primary endpoints were ASAS20, PASI and ACR20, respectively, (Table 1).

**Figure 1:**
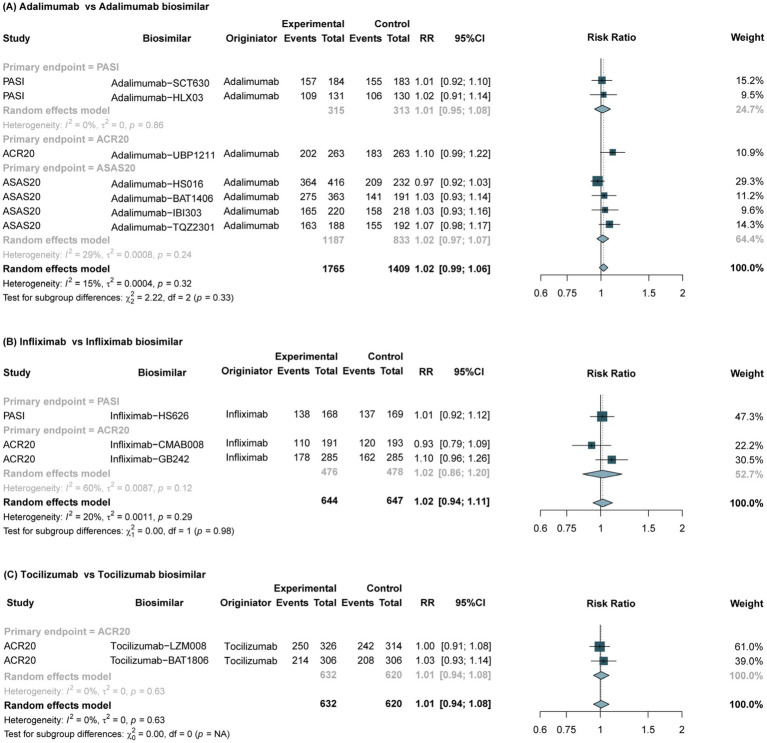
Forest plots of the primary endpoints between biosimilars and reference drugs for IMIDs. IMIDs, Immune-Mediated Inflammatory Diseases; RR, risk ratio; CI, confidence interval; ACR20, American College of Rheumatology 20% improvement criteria; PASI, Psoriasis Area and Severity Index; ASAS20, Assessment of Spondylo Arthritis international Society 20% improvement criteria.

The overall pooled results showed no significant differences between adalimumab biosimilars and the originators in the primary endpoints (Figure 1A). Subgroup analysis showed that no significant differences in the primary endpoint of PASI rate (RR, 1.01, 95% CI, 0.95–1.08; *p* = 0.86), ACR20 rate (RR, 1.10; 95% CI, 0.99–1.22) or ASAS20 rate (RR, 1.02; 95% CI, 0.97–1.07; *p* = 0.24) between adalimumab biosimilars and the reference drugs were observed among patients with RA and AS, respectively. For secondary endpoints, the assessments of ASAS40 and PASI75 were consistent with the primary endpoints. Furthermore, no significant differences were found in safety outcomes (TEAE, Drug-related TEAE, SAE, hypersensitivity, drug interruption, and drug discontinuations), immunogenicity (ADA and Nabs) outcomes or disease subgroups between the Adalimumab biosimilars and their reference drugs (Supplementary Tables S4, S7, S8; Supplementary Figures S2–S6).

Two cohort studies (40, 41) with low risk of adalimumab biosimilars were for Rheumatoid arthritis and Inflammatory Bowel Diseases, respectively (Supplementary Table S6). The results showed that no differences in clinical benefit between adalimumab biosimilars and reference drugs were observed among real-world studies.

#### 3.2.2 Infliximab biosimilars vs. infliximab

Three RCTs compared the Infliximab originator with biosimilars: two studies involved patients with RA (35, 38), and one study involved patients with PP (34). The primary endpoints were ACR20 and PASI, respectively (Table 1).

The meta-analysis of 3 RCTs showed that the primary endpoint of Infliximab biosimilars was comparable to that of the reference drugs in the treatment of IMIDs (RR, 1.02; 95% CI, 0.94–1.11; *p* = 0.98) (Figure 1B). No significant differences in the primary endpoint of ACR20 rate (RR, 1.02; 95% CI, 0.86–1.20; *p* = 0.12) or PASI rate (RR, 1.01; 95% CI, 0.92–1.12) between infliximab biosimilars and the reference drugs were observed among patients with RA and AS, respectively. The assessments of secondary endpoints (ACR50 and PASI 75) were consistent with the primary endpoint. Furthermore, no significant differences were found in safety (TEAE, SAE and other outcomes), immunogenicity endpoints (ADA and Nabs) and and disease subgroups between the Infliximab biosimilars and their reference drugs (Supplementary Tables S4, S7, S8; Supplementary Figures S2–S6).

There were two cohort studies (42, 43) of infliximab biosimilars for inflammatory bowel disease, and both of two cohort studies were low risk (Supplementary Table S6). The results showed no significant differences in efficacy and safety between the Infliximab biosimilar and the reference drug.

#### 3.2.3 Tocilizumab biosimilars vs. tocilizumab

The meta-analysis of 2 RCTs (36, 39) revealed no significant difference in the primary efficacy endpoint (ACR20) between tocilizumab biosimilars and the reference drug in the treatment of RA, (RR, 1.01; 95% CI, 0.94–1.08; *p* = 0.63) (Figure 1C). The assessments of secondary efficacy were consistent with the primary endpoints. Furthermore, no significant differences were found in safety and immunogenicity outcomes between the tocilizumab biosimilars and the reference drugs (Supplementary Tables S7, S8).

### 3.3 Cost and uptake of Biosimilars vs. reference drugs

**Figure 2:**
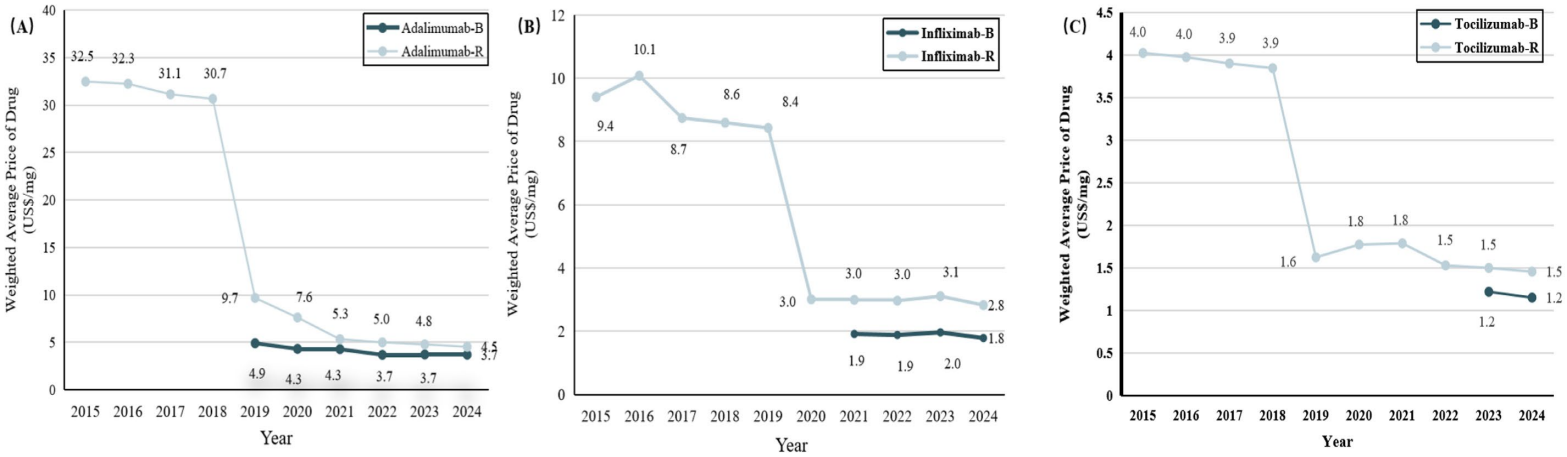
Trends in weighted average price (WAP) per unit for biosimilars and reference drugs for IMIDs between 2015 and 2024. **(A)** Adalimumab versus adalimumab biosimilars. **(B)** Infliximab versus infliximab biosimilars. **(C)** Tocilizumab versus tocilizumab biosimilars. IMIDs, Immune-Mediated Inflammatory Diseases; B, biosimilars; R, reference drugs.

The median weighted average price (WAP) of IMIDs biosimilars and their reference drugs from 2015 to 2024 is shown in Figure 2; Supplementary Tables S9, S10. Adalimumab and tocilizumab were listed on China’s National Reimbursement Drug List (NRDL) in 2019, since then their prices have dropped significantly. Infliximab was included in the NRDL in 2020. As of November 1, 2024, NMPA has approved 7, 5, and 3 biosimilars for adalimumab, infliximab, and tocilizumab, respectively. The estimated median WAP was 82% of the reference drugs for adalimumab biosimilars, 63% for infliximab biosimilars, and 79% for tocilizumab biosimilars.

**Figure 3:**
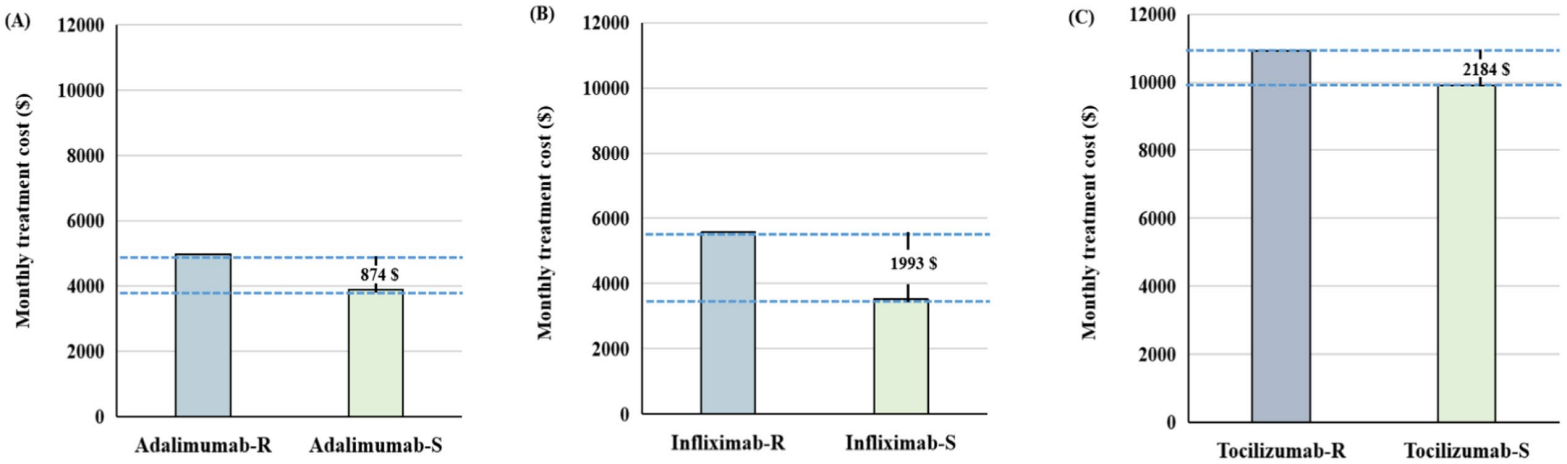
The difference in monthly treatment costs between biosimilars and reference drugs for patients with rheumatoid arthritis in 2024. **(A)** Adalimumab versus adalimumab biosimilars. **(B)** Infliximab versus infliximab biosimilars. **(C)** Tocilizumab versus tocilizumab biosimilars. IMIDs, Immune-Mediated Inflammatory Diseases; B, biosimilars; R, reference biologics.

In terms of monthly treatment costs, IMIDs biosimilars showed varying degrees of cost savings across different indications (Figure 3; Supplementary Table S6). Taking RA as an example, infliximab biosimilars had the lowest monthly treatment cost of $3,430, representing a monthly savings of $1,993 compared to the originator. Tocilizumab was the last to enter the market, with the biosimilar priced at $8,736, resulting in a savings of $2,184, compared to the originator (Figure 3).

**Figure 4:**
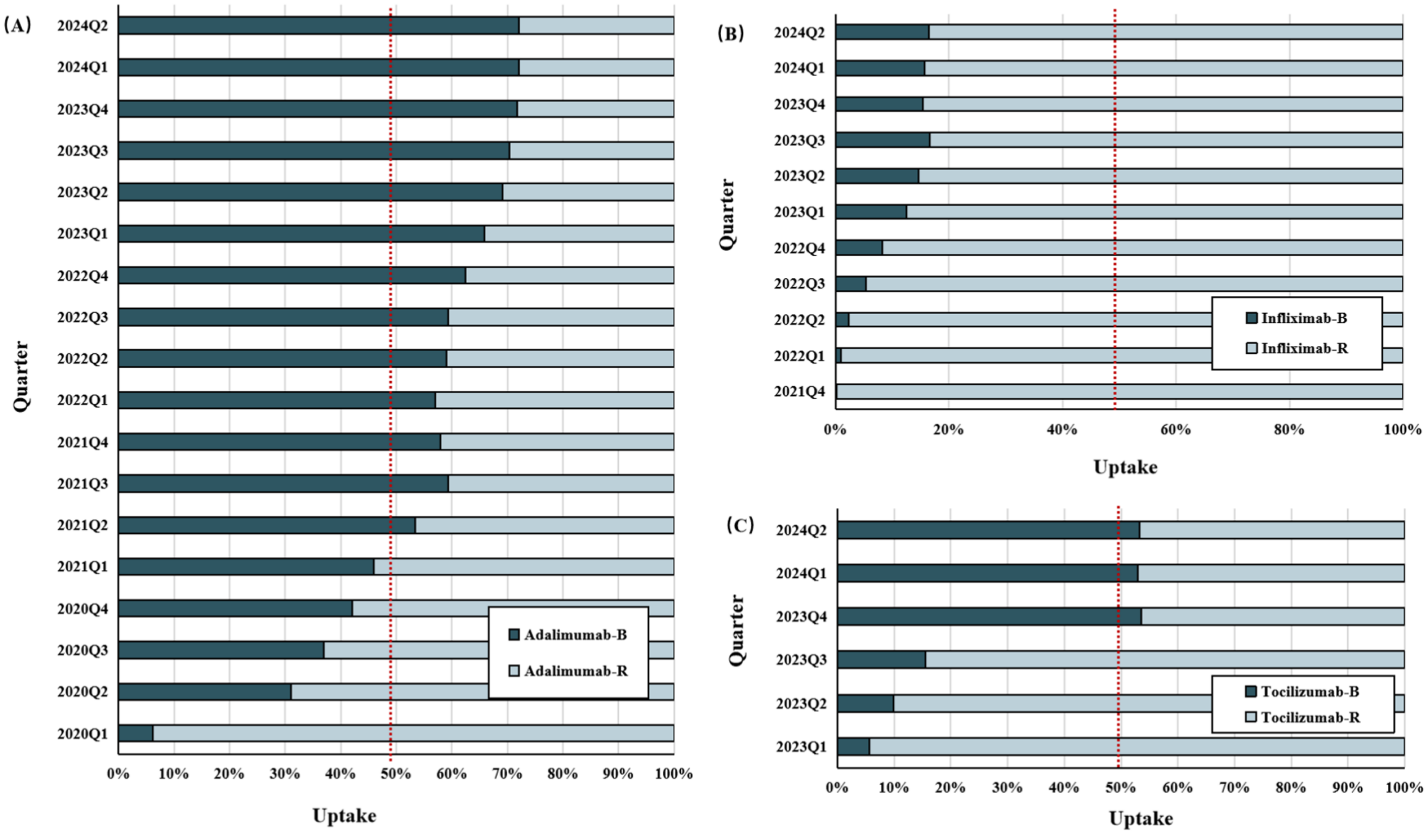
The uptake rate of biosimilars versus reference drugs since biosimilars’ entry into the Chinese market (As of July 2024). **(A)** Adalimumab versus adalimumab biosimilars. **(B)** Infliximab versus infliximab biosimilars. **(C)** Tocilizumab versus tocilizumab biosimilars. IMIDs, Immune-Mediated Inflammatory Diseases; B, biosimilars; R, reference biologics.

IMIDs biosimilars have shown steady uptake growth since the first biosimilar entered the market, despite varying growth rates compared to the originator (Figure 4; Supplementary Table S11). In China, the uptake was highest for adalimumab (36%) and lowest for infliximab (2%) 1 year after market entry. The latest data for 2024 showed that biosimilars of adalimumab, infliximab, and tocilizumab had a market share of 72.1, 16.5, and 53.3%, respectively.

### 3.4 Substitution scenarios of biosimilars

For the first scenario, the results indicated that, in 2023, the use of biosimilars could save $15.9 M for adalimumab, $5.2 M for infliximab, and $0.9 M for tocilizumab. Moreover, substituting biosimilars for reference biologics would allow to support an additional 4,643, 1,527, and 1,071 patients annually for adalimumab, infliximab, and tocilizumab, respectively. Additionally, for the second scenario, with 100% biosimilar substitution, savings would increase to $22.98 M for adalimumab, $33.83 M for infliximab, and $3.82 M for tocilizumab, enabling treatment for 6,700, 9,863, and 4,373 more patients, respectively(Supplementary Table S12).

## 4 Discussion

This study was the first systematic analysis evaluating the relationship between the clinical benefits (efficacy/effectiveness, safety and immunogenicity) and the costs of biosimilars for autoimmune diseases in China. Additionally, the uptake of these biosimilars and their impact on treatment costs were also evaluated. The outcomes of the study showed that all RCTs of IMIDs biosimilars were conducted using double-blinded design. Among these RCTs, 10 (83.3%) were classified as low risks, while 2 (17.0%) had uncertain risk. Most of the biosimilars followed an equivalence trial design, except for Infliximab-GB242, which employed a non-inferiority design despite meeting equivalence thresholds and showing similar safety and immunogenicity. Non-inferiority trials are smaller than equivalence trials but cannot rule out the possibility that a biosimilar may have increased activity, potentially leading to more adverse events or suggesting it could be considered a biobetter (44). Given the risks of non-inferiority design, the FDA, EMA, and NMPA all recommend using an equivalence design in general, while non-inferiority designs should be chosen with caution (45).

This study identified 12 head-to-head RCTs involving 5,717 patients with IMIDs that compared the effects of biosimilars of adalimumab, infliximab, and tocilizumab to their reference drugs. Summary estimates met the prespecified criteria for equivalence based on 12 trials for primary endpoints (including ACR20, ASAS20, and PASI). In addition, our analysis of secondary outcomes revealed no significant differences between biosimilars and originators in PASI75, ASAS40 and ACR50. Safety outcomes, including TEAE, drug-related TEAE, SAE, hypersensitivity reactions, drug interruptions and discontinuations were also evaluated. Biosimilars demonstrated a comparable level of safety to the originators. In terms of immune response, biosimilars did not show a higher incidence of immunogenicity, which was consistent with previous studies (13, 46). This evidence supports the approval of these biosimilars in China with similar clinical benefits to the reference drugs. However, when we conducted further subgroup analyses of diseases, it was found that in the treatment of RA, IMIDs biosimilar may have higher immunogenicity than originators (Nabs, *p* = 0.006) (Supplementary Table S8). This also suggests that post-market immunogenicity monitoring should be strengthened in the future.

While RCTs are valuable, they may have limitations due to their short study duration and strict patient inclusion and exclusion criteria. Real-world studies provide important additional evidence by assessing patient clinical benefit through the data from real clinical patient encounters. In other countries such as the US, EU and Japan, real-world studies are widely used as supplementary evidence to evaluate the clinical benefits of biosimilars in various fields, including oncology, rheumatoid arthritis (28, 40–45, 47–52). Our study included 4 real-world studies of IMIDs biosimilars conducted worldwide, all of which showed that biosimilars have similar clinical benefits to originators (Supplementary Table S4). Real-world studies on oncology biosimilars are currently conducted in China. Previous studies have shown that three retrospective real-world studies conducted in China on bevacizumab were instrumental in supporting the approval for use in combination with platinum-based chemotherapy regimens as a first-line treatment for advanced non-squamous non-small cell lung cancer (53, 54). Given the high malignancy of cancer and the potential risks associated with treatment efficacy differences leading to disease progression, the CDE has emphasized the need for post-market immunogenicity monitoring in its guidelines (51–53). Strengthening real-world evidence for IMID biosimilars regulatory will be essential moving forward.

Indication extrapolation of biosimilars from the reference drugs may contribute to improving patient accessibility and accelerating the uptake of biosimilars. The US, the EU, Japan and other countries have established extrapolation policies, while adopting patent and data exclusivity systems to maintain the balance between innovation and imitation (54, 55). China has also issued the Technical Guidelines for Similarity Evaluation and Indication Extrapolation of Biosimilars in 2020 to allow for the extrapolation of biosimilars (56). Our study has shown that biosimilars of adalimumab, infliximab, and tocilizumab have been approved for the majority of originator indications through extrapolation (Supplementary Tables S13, S14). Additionally, our previous study has demonstrated that China’s patent term extension and data exclusivity protection systems are also being actively explored (52). Therefore, it can be expected that China will establish a mechanism to enhance the balance between biosimilars and originators.

The affordability of biologic treatments poses a significant barrier to patient access, highlighting the importance of IMIDs biosimilars in reducing treatment costs (57). A recent study indicated that biosimilars typically ranged from 55 to 90% of the list prices of their reference drugs in the US, Germany, and Switzerland (58). The utilization of adalimumab biosimilars in the US alone could yield healthcare savings of approximately $2.19 billion between 2016 and 2019 (5). In our investigation, we found that IMIDs biosimilars were priced at 63 to 82% of their originators, consistent with the previous studies (58). It should be noted that drug price negotiations in China have been instrumental in reducing the prices of IMIDs biosimilars and their reference drugs (13, 59). According to the China National Health Security Administration, these negotiations have led to an average price reduction of 61.7% for novel drugs (60). Specifically, the prices of reference drugs such as adalimumab, infliximab, and tocilizumab have decreased by 86.0, 69.9, and 63.7%, respectively, following their inclusion in the Chinese health insurance schemes. In this study, biosimilars of adalimumab, infliximab, and tocilizumab were found to offer monthly savings of $874 to $3,322 compared to their originators, which were equivalent to 1.8 to 7.0 times the *per capita* disposable income ($473.6) in China for 2023. This suggests that the use of IMIDs biosimilars could be critical to reducing costs and alleviating the financial burden on patients. Besides, our simulations align with previous studies, showing that biosimilar substitution helps reduce healthcare costs, allowing limited resources to benefit more patients (61).

The substitution effect of biosimilars is influenced by multiple factors. Within the same regulatory region, different uptake rates of IMIDs biosimilars may be related to market competition, cost savings, and extrapolated indications (adalimumab biosimilars have the highest number of approvals and indications, while tocilizumab biosimilars offer the greatest monthly treatment cost savings). The uptake of biosimilars varies significantly across countries, influenced by a variety of factors, such as healthcare negotiations, utilization policies (7, 62, 63). A previous study showed that, within the first year of launch, adalimumab biosimilars achieved market shares of 45% in the US, 55% in Germany and 17.5% in Switzerland. In comparison, Denmark showcased a remarkably higher uptake rate, with adalimumab biosimilar reaching a 95.1% market share just 3 months after launch, significantly surpassing other countries. Our study found that the uptake rate of adalimumab biosimilars in China was 42% in the first year after launch, closely aligning with the US but considerably lower than Denmark. The high uptake rate of the Danish model can be mainly attributed to the national tendering and procurement strategies, alternative treatment recommendations issued by the Danish Medicines Agency, and effective multi-stakeholder cooperation. These strategies were similar to China’s national volume-based procurement (NVBP), which has also yielded favorable outcomes in insulin. Therefore, accelerating the uptake of IMIDs biosimilars through China’s national volume-based procurement (NVBP) and multistakeholder consensus should be considered in China. Additionally, the uptake rate of biosimilars can be influenced by the preferences of both patients and clinicians. Further efforts to enhance the understanding of biosimilars among physicians and patients are needed in China.

## 5 Limitation

This study has certain limitations. Firstly, while the study makes thorough use of RCT data, the absence of RWE weakens its generalizability. Real-world studies are crucial to validate RCT findings, particularly in understanding long-term safety and effectiveness, especially for biologics and biosimilars. Although RWE from other countries has been included, the lack of data from China highlights a critical gap, underscoring the need for future research to address this context-specific evidence deficit. Secondly, our study was limited to the clinical benefits, costs and uptake of IMIDs biosimilars approved in China, making our findings not necessarily applicable to other countries. Third, this study sought to incorporate, to the extent possible, publicly available data from published articles and review reports on IMIDs biosimilars. Despite these efforts, it is possible that some relevant unpublished studies may not have been included. To ensure more comprehensive data inclusion, future studies should expand their search to include gray literature, such as dissertations, clinical trial registries, and preprints, capturing unpublished or ongoing studies.

## 6 Conclusion

Our study revealed that IMID biosimilars in China provide clinical benefits comparable to their reference biologics evidenced by high-quality RCTs and cohort studies with offer significant cost savings in China. In the future, encouraging China’s national volume-based procurement and multi-stakeholder collaboration may help accelerate the substitution of IMIDs biosimilars. Additionally, research on doctors, physicians, and patient behaviors, along with education, may also help ensure rational use and improve accessibility.

## Data Availability

The original contributions presented in the study are included in the article and supplementary material, further inquiries can be directed to the corresponding authors.
