# Supplementary material for: Assessment of clinical benefit, cost and uptake of biosimilars versus reference biologics in immune-mediated inflammatory diseases in China
Source: Front Public Health. 2024 Dec 4;12:1476213. doi: 10.3389/fpubh.2024.1476213 (PMC11652503; doi:10.3389/fpubh.2024.1476213)
Supplement: Supplementary file 1 [file Data_Sheet_1.docx]

**Assessment of Clinical Benefit, Cost and Uptake of Biosimilars versus Reference Biologics in Immune-Mediated Inflammatory Diseases in China**

Xin Du^1#^, Xingxian Luo^2#^, Qixiang Guo^3,4^, Xiaomeng Jiang^3,4^, Ziling Su^5^, Weiting Zhou^6^, Zhongjian Wang^7^, Jiarun Li^1^, Yue Yang^3, 4*^, Yi, Zhang^1*^.

1. Vanke School of Public Health, Tsinghua University, Beijing, China.

2. Department of Pharmacy, Peking University People's Hospital, Beijing, China.

3. School of Pharmaceutical Sciences, Tsinghua University, Beijing, China.

4. Key Laboratory of Innovative Drug Research and Evaluation, National Medical Products Administration, Beijing, China.

5. School of Pharmaceutical Sciences, Sun Yat-sen University, Guangzhou, China.

6. School of Pharmacy, Massachusetts College of Pharmacy and Health Sciences University, Boston, United States.

7. Pharnexcloud Digital Technology (Chengdu) Co., Ltd, Chengdu 610093, China.

^#^ Co-first author:

Xin Du, Vanke School of Public Health, Tsinghua University, Beijing, China. (e-mail:du-x23@mails.tsinghua.edu.cn)

Xingxian Luo, School of Pharmaceutical Sciences, Tsinghua University, Beijing, China. (e-mail: [luoxingxian@pkuph.edu.cn](mailto:luoxingxian@pkuph.edu.cn)).

*Corresponding authors:

Yue Yang, School of Pharmaceutical Sciences, Tsinghua University, Beijing, China. (e-mail: yanghappy[@tsinghua.edu.cn](mailto:huanglin@pkuph.edu.cn); TEL: +8613126800315).

Yi Zhang, Vanke School of Public Health, Tsinghua University, Beijing, China. (e-mail: yi_zhang@mail.tsinghua.edu.cn; TEL: +8615001089920).

**Supplementary Appendix**

[eAppendix 1 A detailed description of the eligibility and study selection criteria. 0](#_Toc182435630)

[eAppendix 2. Price, cost and uptake of biosimilars and reference drugs in China. 1](#_Toc182435631)

[eTable S1. The IMIDs biosimilars approved in China, the EU and the US. 0](#_Toc182435632)

[eTable S2. Comparsion of regulatory requirements for biosimilars by the FDA, EMA, PMDA and NMPA. 1](#_Toc182435633)

[eTable S3. The number of approved and in-development IMIDs biosimilars in China. 0](#_Toc182435634)

[eTable S4. Characteristics of the included cohort studies. 1](#_Toc182435635)

[eTable S5. Results of the risk of bias assessment for randomized clinical trials using the Cochrane Collaboration's Tool. 2](#_Toc182435636)

[eTable S6. Results of the risk of bias assessment for cohort studies using the Newcastle-Ottawa Risk of Bias Assessment Tool. 3](#_Toc182435637)

[eTable S7. Subgroup analysis comparing IMIDs biosimilars with reference drugs derived from randomized clinical trials. 4](#_Toc182435638)

[eTable S8. Subgroup analysis comparing IMIDs biosimilars with reference drugs by indication. 6](#_Toc182435639)

[eTable S9. Weighted mean price per unit of Adalimumab, Infliximab, Tocilizumab, and their biosimilars between 2015 and 2024. 8](#_Toc182435640)

[eTable S10. Weighted average monthly treatment costs of Adalimumab, Infliximab, Tocilizumab, and their biosimilars for patients with Rheumatoid Arthritis in 2024. 9](#_Toc182435641)

[eTable S11. Uptake rates of Adalimumab, Infliximab, Tocilizumab, and their biosimilars between 2015 and 2024. 11](#_Toc182435642)

[eTable S12. Savings and additional patient benefits under various biosimilar substitution scenarios. 13](#_Toc182435643)

[eTable S13. Comparison of approved indications for IMIDs originators and biosimilars by the NMPA. 15](#_Toc182435644)

[eTable S14. Reimbursable IMIDs indications for Adalimumab, Infliximab, and Tocilizumab biosimilars and reference drugs in China’s National Reimbursement Drug List (As of January 1, 2024). 17](#_Toc182435645)

[eFigure S1. Flowchart of included studies for the systematic review. 18](#_Toc182435646)

[eFigure S2. Forest plots for the american college of rheumatology 20% response criteria (ACR20) for primary endpoints at 24 weeks after treatments with biosimilars and their reference drug. 19](#_Toc182435647)

[eFigure S3. Forest plots for the assessment of spondylo arthritis international society (ASAS) for primary endpoints at 24 weeks after treatments with biosimilars and their reference drug. 20](#_Toc182435648)

[eFigure S4. Forest plots of psoriasis area and severity index（PASI）for primary endpoints after treatments with biosimilars and their reference drug. 21](#_Toc182435649)

[eFigure S5. Subgroup analysis of adalimumab biosimilar based on risk of bias assessment results. 22](#_Toc182435650)

[eFigure S6. Subgroup analysis of torizumab biosimilar based on risk of bias assessment results. 23](#_Toc182435651)

[Reference 24](#_Toc182435652)

**eAppendix 1 A detailed description of the eligibility and study selection criteria.**

Participants: Patients diagnosed with immune-mediated inflammatory diseases (IMIDs) using validated and established international criteria. No restrictions were imposed on the baseline IMIDs severity, age, sex, line of treatment, or any other major demographic characteristics of the patients.

Interventions and comparators: The intervention group included any biosimialrs of adalimumab, tocilizumab, and infliximab. The comparators of interest were their respective reference biologics. These three main biologics were chosen because they have the highest number of approved biosimilars for treating IMIDs in China (as of November 1, 2024).^1^ Like previous studies, we did not restrict dosages, treatment duration, patient numbers, or the use of combined therapies and co-treatments.^2,3^

Type of study: Eligible trials included randomized controlled trials (RCTs) and cohort studies (comparative studies of biosimilars and originator drugs). We incorporated all trials that compared biosimilars with originator drugs, irrespective of the statistical design type (superiority, equivalence, or non-inferiority). Trials without comparators and non-comparative studies (e.g. reviews, expert commentaries, editorials, and clinical guidelines) were excluded. For trials with a two-part study design, we considered only the results from the first period (biosimilarity) to avoid carry-over effects.

**eAppendix 2. Price, cost and uptake of biosimilars and reference drugs in China.**

Price and cost: China has a three-tier healthcare delivery system, with healthcare institutions and providers operating at different levels: at the county, township, and village levels in rural areas, and at the municipal, district, and community levels in urban areas.^4^ Drug procurement approaches, which encompass national centralized bulk-purchasing, provincial bidding and purchasing, and other methods, contribute to price variations across regions. Therefore, we extracted the winning bid price from each province to calculate the annual weighted average price of biosimilars per milligram. Meanwhile, we adjusted the price of biosimilars to reflect 2024 values, accounting for inflation. Finally, we converted these into US dollars based on the 2024 exchange rate between the Chinese Yuan (RMB) and the US Dollar (USD) in 2023, which was 1 USD to 7.1 RMB.^5^

In addition, we calculated the monthly treatment costs for both reference biologics and biosimilars individually, using information from the drug labels. The annual treatment cost was determined by multiplying the dose administered to a patient over one year (52 weeks) by the unit price. Subsequently, we calculated the average monthly treatment cost over a 12-month period.

Uptake: In China, healthcare institutions are categorized into primary healthcare institutions, secondary comprehensive hospitals and tertiary comprehensive hospitals. Normally, the higher-level institutions provide more sophisticated treatments. In this study, we utilized national hospital sales volume data from the Pharnexcloud database module,^3^ which covered the sales volumes of cancer biosimilars in secondary and tertiary hospitals nationwide, as no public database was available for drug sales volumes. We separately extracted the sales volume data of biosimilars and reference drugs in China for each quarter. The uptake of biosimilar was defined as the ratio of biosimilar sales volume to the total sales volume (including both biosimilars and reference drugs). The dataset spans from the introduction of biosimilars to the market until July 1, 2024.

**eTable S1. The IMIDs biosimilars approved in China, the EU and the US.**

| Biosimilar | China | | EU | | US | |
| --- | --- | --- | --- | --- | --- | --- |
|  |  | |  | |  | |
|  | Reference product | Biosimilars | Reference product | Biosimilars | Reference product | Biosimilars |
| Adalimumab | Humira  (2010) | HS016（2019）  IBI303（2020）  HLX03（2020）  BAT1406（2021） UBP1211（2022）  TQ-Z2301（2022）  SCT630（2023） | Humira (2003) | Imraldi (2017)  Amgevita (2017)  Hefiya (2018)  Hyrimoz (2018)  Hulio (2018)  Idacio (2019)  Amsparity (2020)  Yuflyma (2021)  Hukyndra (2021)  Libmyris (2021) | Humira (2002) | Amjevita(2016)  Cyltezo(2017)  Hyrimoz(2018)  Abrilada(2019)  Hadlima(2019)  Hulio(2020)  Yusimry (2021)  Idacio (2022)  Yuflyma (2023)  Simlandi(2024) |
| Infliximab | Remicade  （2006） | SCT630（2021）  HS626（2021）  GB242（2022）  CT-P13（2023） | Remicade (1999) | Remsima（2013）  Inflectra（2013）  Flixabi（2016）  Zessly（2018） | Remicade (1998) | Inflectra(2016)  Ixifi (2017)  Renflexis (2017)  Avsola(2019) |
| Tocilizumab | RoActemra（2013） | BAT1806（2023）  BOW070 (2023)  HS628 (2024)  CMAB806 (2024) | RoActemra (2009) | Tyenne(2023) | ACTEMRA (2010) | BAT1806(2023)  Tyenne(2024) |

Note: Underlining indicates that the biosimilar has been approved for marketing in more than one country.

**eTable S2. Comparsion of regulatory requirements for biosimilars by the FDA, EMA, PMDA and NMPA.**

| Indicator | United States | European Union | Japan | China |
| --- | --- | --- | --- | --- |
| Regulatory statutes or regulations | The FDA manages biosimilar products mainly according to standards set forth in:   1. Section 351of the Public Health Service Act; 2. Food, Drug, and Cosmetic Act. 3. Scientific Considerations in Demonstrating Biosimilarity to a Reference Product. 4. Quality Considerations in Demonstrating Biosimilarity of a Therapeutic Protein Product to a Reference Product. | The EMA manages biosimilar products mainly according to standards set forth in:   1. Directive 2001/83/EC (Community Code on Medicinal Products for Human Use); 2. Guideline on Similar Biological Medicinal Products: Quality, Non-clinical and Clinical Issues. | The PMDA manages biosimilar products mainly according to standards set forth in:   1. Pharmaceutical and Medical Devices Act. 2. Guideline on the Quality, Safety and Efficacy Assurance of Follow-on Biologics. | The NMPA manages biosimilar products mainly according to standards set forth in:   1. Pharmaceutical Administration Law of the People's Republic of China. 2. Guideline on the Similarity Assessment and Extrapolation of Indications for Biosimilars. |
| Review requirements for clinical similarity | The FDA recommends using equivalence trials over non-inferiority trials for comparing biosimilars to reference biologics. This preference stems from the need to establish that a biosimilar product not only matches the reference product in efficacy but also closely aligns in safety and immunogenicity, with no clinically meaningful differences. | The EMA recommends using equivalence trials over non-inferiority trials for biosimilars. According to EMA guidelines, equivalence trials are considered more suitable for demonstrating biosimilarity, especially for therapeutic protein products, as they ensure that the biosimilar’s efficacy and safety are neither inferior nor superior to the reference product. | The PMDA favors equivalence designs but allows non-inferiority trials under certain conditions. The PMDA’s Guideline on the Quality, Safety and Efficacy Assurance of Follow-on Biologics encourages equivalence trials but permits non-inferiority designs when there is sufficient scientific justification. | The NMPA recommends that clinical comparative effectiveness trials usually adopt an equivalence design, and a non-inferiority design should be carefully selected with a reasonable threshold. For a non-inferiority design, the degree of variation in the clinical efficacy of the reference drug in the comparative trial should be considered to evaluate the similarity between the candidate drug and the reference drug. |
| Indication Extrapolation | Primary consideration is given to core patents, with a more limited impact from patents on indications; Exclusivity periods have not been established. | Impact of core and indication patents; Impact of exclusivity period (especially for the 7-year orphan drug designation). | Impact of core and indication patents; Impact of exclusivity period. | Impact of core and indication patents; Impact of exclusivity period. |
| Requirement for Real World Data | The FDA encourages the use of Real-World Data (RWD) to assess the performance of biosimilars in real-world settings, particularly for detecting rare adverse events. However, the requirements for indication extrapolation are stricter. | The EMA recommends the pharmacovigilance and Risk Management Plans, using RWD to continuously assess the safety of biosimilars. It requires documenting patient characteristics, treatment regimens, and clinical outcomes in real-world studies to support the justification for indication extrapolation. | The PMDA supports using RWD for post-marketing surveillance of biosimilars, but is cautious about indication extrapolation. It also recommends using RWD to supplement clinical trial data, but indication extrapolation must be based on strong scientific evidence, especially when there are significant differences in patient characteristics, disease pathology, or drug efficacy across indications. The PMDA prefers clear clinical data for each indication to minimize risks. | The NMPA encourages the use of real-world data to conduct more comprehensive evaluations of a drug's effectiveness, safety, usage, and economic benefits in real-world clinical practice, and to continuously adjust decisions based on real-world evidence. |
| Reimbursement policy | Determined by individual insurers | Determined by national authorities | Determined centrally by Japan’s Central Social Medical Insurance Council of the Ministry of Health, Labour, and Welfare | Determined by China National Healthcare Security Administration |
| Switching patients from a reference biologic to a biosimilar | Not permitted unless the biosimilar has been formally designated as interchangeable biosimilar by FDA | No regulatory designation for interchangeability | Permitted under the PMDA’s Guideline for the quality, safety, and efficacy assurance of follow-on biologics (revised Feb 2, 2020) | No regulatory designation for interchangeability |
| Pharmacist substitution of a reference biologic with a biosimilar | Not permitted unless FDA has designated the biosimilar as interchangeable with the reference biologic, and even if it has 45 US states do not permit substitution unless patient consent, doctor consent, or both have been documented | Permitted in several EU countries | Not permitted | Not permitted |

Note: NA, not applicable; FDA, US Food and Drug Administration; EMA, European Medicines Agency; PMDA, Japanese Pharmaceuticals and Medical Devices Agency; NMPA, Chinese National Medical Products Administration.

**eTable S3. The number of approved and in-development IMIDs biosimilars in China.**

| Biosimilar | Phase I | Phase III | NDA review | Approval |
| --- | --- | --- | --- | --- |
| Adalimumab | 4 | 3 | 1 | 7 |
| Infliximab | 0 | 0 | 0 | 4 |
| Tocilizumab | 2 | 0 | 0 | 4 |

Note: NDA, new drug applications.

**eTable S4. Characteristics of the included cohort studies.**

| First Author | Year | Country | Sex(M/F) | Disease types | Originator | Biosimilar | No. patients in originator | No. patients in biosimilar | Main efficacy outcome (originator vs biosimilar) | Safety outcome (originator vs biosimilar) | Study durationa (month) |
| --- | --- | --- | --- | --- | --- | --- | --- | --- | --- | --- | --- |
| Adalimumab vs adalimumab biosimilar | | | | | | | | | | | |
| Popescu CC, et al. | 2022 | Romania | NA | Rheumatoid arthritis | Adalimumab | Adalimumab biosimilar | 213 | 228 | 6-month remission rates: DAS28-ESR: 50/213 vs 35/228 (*P*=0.148) DAS-CRP: 69/228 vs 74/228 (*P*=0.686) | TEAE: 173/223% vs 43/228 | 6 |
| Mocci G, et al. | 2022 | Italy | 67/67 | Inflammatory Bowel Diseases | Adalimumab | Adalimumab- GP2017 | 72 | 62 | Remission: 54/72 vs 51/63 (P=0.311) | TEAE: 4/72 vs 1/62 | 12 |
| Infliximab vs infliximab biosimilar | | | | | | | | | | | |
| Kumar P, et al. | 2022 | India | NA | Inflammatory bowel disease | Infliximab | Infliximab biosimilar | 102 | 35 | Crohn’s disease: 36/63 vs 13/19 (*P*=0.38); Ulcerative colitis: 22/39 vs 10/16 (*P*=0.68) | TEAE of Crohn’s disease: 20/63 vs 3/19; TEAE of Ulcerative colitis: 7/39 vs 3/16; | 24-36 |
| Smith JT, et al. | 2021 | US | 1590/1616 | Inflammatory bowel disease | infliximab | Infliximab-dyyb | 870 | 2336 | Composite effectiveness outcome: 107/870 vs 379/2336 (*P*=0.06) | Any infection: 219/870 vs 687/2336 (P=0.95) | 11 |

Note: TEAE, treatment emergent adverse event; DAS, disease activity score.

**eTable S5. Results of the risk of bias assessment for randomized clinical trials using the Cochrane Collaboration's Tool.**

| First Author | Year | Random sequence generation (selection bias) | Allocation concealment (selection bias) | Blinding of participants and personnel (performance bias) | Blinding of outcome assessors (performance bias) | Incomplete outcome data  (attrition bias) | Selective outcome reporting  (reporting bias) | Other potential bias | Overall  assessment:  risk of bias |  |
| --- | --- | --- | --- | --- | --- | --- | --- | --- | --- | --- |
|  |  |  |  |  |  |  |  |  |  |  |
| Adalimumab biosimilars and Adalimumab | | | | | | | | | |  |
| Yu et al^6^ | 2022 | ? | ➕ | ➕ | ➕ | ➕ | ➕ | ➕ | low |  |
| Li et al^7^ | 2022 | ➕ | ➕ | ➕ | ➕ | ➕ | ➕ | ➕ | low |  |
| Xu et al^8^ | 2020 | ➕ | ➕ | ➕ | ➕ | ➕ | ➕ | ➕ | low |  |
| Su et al^9^ | 2022 | ➕ | ➕ | ➕ | ➕ | ➕ | ➕ | ➕ | low |  |
| Cai et al^10^ | 2020 | ➕ | ➕ | ➕ | ➕ | ➕ | ➕ | ➕ | low |  |
| Tu et al^11^ | 2019 | ? | ? | ➕ | ? | ➕ | ➕ | ➕ | unclear |  |
| TopAlliance^12^ (sponsor) | 2020 | ➕ | ➕ | ➕ | ? | ➕ | ➕ | ➕ | low |  |
| Infliximab biosimiars and Infliximab | | | | | | | | | |  |
| Liu et al^13^ | 2022 | ➕ | ➕ | ➕ | ➕ | ➕ | ➕ | ➕ | low |  |
| Hisun (sponsor)^14^ | 2021 | ➕ | ➕ | ➕ | ? | ➕ | ➕ | ➕ | low |  |
| Ye et al^15,16^ | 2021 | ➕ | ➕ | ➕ | ➕ | ➕ | ➕ | ➕ | low |  |
| Tocilizumab biosimilar and Tocilizumab | | | | | | | | | |  |
| Leng et al^17,18^ | 2023 | ? | ? | ➕ | ? | ➕ | ➕ | ➕ | unclear |  |
| Lizhu (sponsor)^18^ | 2023 | ➕ | ➕ | ➕ | ? | ➕ | ➕ | ➕ | low |  |

Note: RCTs, randomized controlled trials.

**eTable S6. Results of the risk of bias assessment for cohort studies using the Newcastle-Ottawa Risk of Bias Assessment Tool.**

| First Author | Year | Representativeness of the exposed cohort | Selection of the non-exposed cohort | Ascertainment of exposure | Demonstration that outcome of interest was not present at baseline | Comparability of cohorts on the basis of the design or analysis | Assessment of outcomes | Was follow-up long enough for outcomes occur | Adequacy of follow up of cohorts | Total scores^a^ (risk of bias) |
| --- | --- | --- | --- | --- | --- | --- | --- | --- | --- | --- |
| Popescu CC, et al. | 2022 | 1 | 1 | 1 | 1 | 0 | 1 | 1 | 1 | 7 |
| Mocci G, et al. | 2022 | 1 | 1 | 1 | 1 | 1 | 1 | 1 | 0 | 7 |
| Kumar P, et al. | 2022 | 1 | 1 | 1 | 1 | 1 | 1 | 1 | 0 | 7 |
| Smith JT, et al. | 2021 | 1 | 1 | 1 | 1 | 1 | 1 | 1 | 1 | 8 |

Note: The green, yellow and red colours indicates low risk, some concerns and high risk of bias, respectively;^a^ Total scores (risk of bias): scoring from 7-9: high quality; Scoring from 4-6: high risk of bias; Scoring from 0-3: very high risk of bias.

**eTable S7. Subgroup analysis comparing IMIDs biosimilars with reference drugs derived from randomized clinical trials.**

| Subgroup | No.of studies | Biosimilar, n | Originator, n | RR (95%CI) | Test of heterogeneity | | *P* value |
| --- | --- | --- | --- | --- | --- | --- | --- |
|  |  |  |  |  | I^2^ (%) | *P* value |  |
| Adalimumab biosimilar vs Adalimumab | | | | | | | |
| Primary endpoint | | | | | | | |
| ASAS20 | 4 | 1187 | 833 | 1.01 (0.97,1.05) | 24 | 0.24 | 0.559 |
| PASI | 2 | 315 | 313 | 1.01 (0.95,1.08) | 0 | 0.86 | 0.728 |
| ACR20 | 1 | 263 | 263 | 1.10 (0.99,1.22) | NA | NA | 0.062 |
| Secondary endpoint | | | | | | | |
| ASAS40 | 3 | 824 | 642 | 0.99 (0.92,1.06) | 0 | 0.43 | 0.680 |
| PASI75 | 2 | 315 | 313 | 1.03 (0.97,1.10) | 0 | 0.63 | 0.313 |
| Safety outcome | | | | | | | |
| TEAE | 4 | 933 | 847 | 0.99 (0.95,1.02) | 0 | 0.44 | 0.436 |
| Drug-related TEAE | 3 | 802 | 533 | 0.97 (0.89,1.06) | 0 | 0.87 | 0.498 |
| SAE | 5 | 1099 | 746 | 0.80 (0.42,1.54) | 37 | 0.18 | 0.5036 |
| hypersensitivity | 1 | 166 | 83 | 1.00 (0.42,2.38) | NA | NA | 1.000 |
| drug interruption | 2 | 408 | 410 | 1.06 (0.41,2.75) | 50 | 0.16 | 0.909 |
| drug discontinuations | 1 | 191 | 190 | 1.28 (0.49; 3.36) | NA | NA | 0.618 |
| Immunogenicity outcome | | | | | | | |
| ADA | 7 | 1741 | 1298 | 1.00 (0.95,1.04) | 8 | 0.37 | 0.852 |
| Nabs | 6 | 1330 | 1058 | 0.93 (0.82,1.05) | 0 | 0.89 | 0.253 |
| Infliximab biosimilar vs Infliximab | | | | | | | |
| Primary endpoint | | | | | | | |
| ACR20 | 2 | 476 | 478 | 1.02 (0.86,1.20) | 60 | 0.12 | 0.857 |
| PASI | 1 | 168 | 169 | 1.01 (0.92,1.12) | NA | NA | 0.799 |
| Secondary endpoint | | | | | | | |
| ACR50 | 2 | 457 | 462 | 1.11 (0.95,1.31) | 0 | 0.87 | 0.194 |
| PASI75 | 1 | 168 | 169 | 1.01 (0.92,1.12) | NA | NA | 0.799 |
| Safety outcome | | | | | | | |
| TEAE | 3 | 643 | 647 | 0.99 (0.94,1.04) | 0 | 0.62 | 0.602 |
| Drug-related TEAE | 1 | 168 | 169 | 0.98 (0.85,1.13) | NA | NA | 0.786 |
| SAE | 2 | 475 | 478 | 0.83 (0.51,1.35) | 0 | 0.98 | 0.444 |
| hypersensitivity | 2 | 475 | 478 | 8.22 (1.51,44.66) | 0 | 0.80 | 0.015 |
| drug interruption | 1 | 283 | 283 | 0.97 (0.46,1.34) | NA | NA | 0.376 |
| drug discontinuations | 1 | 283 | 283 | 1.00 (0.59,1.70) | NA | NA | 1.000 |
| Immunogenicity outcome | | | | | | | |
| ADA | 3 | 643 | 647 | 1.00 (0.93,1.07) | 0 | 0.40 | 0.903 |
| Nabs | 3 | 606 | 647 | 1.15 (0.99,1.35) | 0 | 0.55 | 0.072 |
| Tocilizumab biosimilar vs Tocilizumab | | | | | | | |
| Primary endpoint | | | | | | | |
| ACR20 | 2 | 632 | 620 | 1.01 (0.94,1.08) | 0 | 0.63 | 0.812 |
| Safety outcome | | | | | | | |
| TEAE | 1 | 312 | 309 | 1.02 (0.90,1.14) | NA | NA | 0.797 |
| Drug-related TEAE | 1 | 312 | 309 | 0.97 (0.82,1.14) | NA | NA | 0.721 |
| SAE | 2 | 638 | 623 | 1.18 (0.65,2.16) | 27 | 0.24 | 0.586 |
| Immunogenicity outcome | | | | | | | |
| ADA | 2 | 638 | 623 | 1.49 (1.16,1.92) | 0 | 0.91 | 0.002 |
| Nabs | 2 | 638 | 623 | 1.47 (1.14,1.89) | 0 | 0.91 | 0.003 |

Note: NA, not applicable; PASI, psoriasis area and severity index; PASI75, 75% improvement in PASI score; ACR20, American College of Rheumatology 20% Response Criteria; TEAE, Treatment Emergent Adverse Event; SAE, Serious Adverse Event; ADA, Anti-Drug antibody; Nabs, Neutralizing antibodies.

**eTable S8. Subgroup analysis comparing IMIDs biosimilars with reference drugs by indication.**

| Subgroup | No.of studies | Biosimilar,n | Originator,n | RR (95%CI) | Test of heterogeneity | | *P* value |
| --- | --- | --- | --- | --- | --- | --- | --- |
|  |  |  |  |  | I^2^ (%) | *P* value |  |
| Rheumatoid Arthritis | | | | | | | |
| Primary endpoint | | | | | | | |
| ACR20 | 5 | 1371 | 1361 | 1.03 (0.98, 1.09) | 14 | 0.240 | 0.232 |
| Secondary endpoint | | | | | | | |
| ACR50 | 2 | 457 | 462 | 1.11 (0.95, 1.31) | 0 | 0.865 | 0.130 |
| Safety outcome | | | | | | | |
| TEAE | 3 | 787 | 787 | 1.00 (0.94, 1.07) | 0 | 0.682 | 0.887 |
| Drug-related TEAE | 1 | 312 | 309 | 0.97 (0.82, 1.14) | NA | NA | 0.708 |
| SAE | 5 | 1113 | 1101 | 0.99 (0.69, 1.40) | 0 | 0.488 | 0.938 |
| Drug interruption | 1 | 283 | 283 | 0.79 (0.46, 1.34) | NA | NA | 0.376 |
| Immunogenicity outcome | | | | | | | |
| ADA | 5 | 1376 | 1364 | 1.10 (0.98, 1.22) | 58 | 0.048 | 0.116 |
| Nabs | 5 | 1339 | 1364 | 1.21 (1.05, 1.38) | 14 | 0.326 | 0.006 |
| Ankylosing Spondylitis | | | | | | | |
| Primary endpoint | | | | | | | |
| ASAS20 | 4 | 1187 | 833 | 1.02 (0.97, 1.07) | 29 | 0.237 | 0.556 |
| Secondary endpoint | | | | | | | |
| ASAS40 | 3 | 824 | 642 | 0.98 (0.92, 1.05) | 0 | 0.448 | 0.659 |
| Safety outcome | | | | | | | |
| TEAE | 3 | 636 | 634 | 0.99 (0.94, 1.04) | 0 | 0.565 | 0.721 |
| Drug-related TEAE | 2 | 636 | 450 | 0.96 (0.88, 1.05) | 0 | 0.899 | 0.408 |
| SAE | 3 | 802 | 533 | 0.74 (0.25, 2.20) | 68 | 0.044 | 0.590 |
| Drug interruption | 2 | 408 | 410 | 1.06 (0.41, 2.75) | 50 | 0.156 | 0.909 |
| Immunogenicity outcome | | | | | | | |
| ADA | 4 | 1187 | 833 | 0.99 (0.93, 1.05) | 17 | 0.306 | 0.692 |
| Nabs | 3 | 824 | 642 | 0.91 (0.78, 1.07) | 0 | 0.770 | 0.259 |
| Plaque Psoriasis | | | | | | | |
| Primary endpoint | | | | | | | |
| ASAS40 | 3 | 483 | 482 | 1.04 (0.98, 1.10) | 5.3 | 0.348 | 0.232 |
| Secondary endpoint |  |  |  |  |  |  |  |
| PASI75 | 3 | 483 | 482 | 1.02 (0.97, 1.08) | 0 | 0.797 | 0.446 |
| Safety outcome | | | | | | | |
| TEAE | 3 | 465 | 382 | 0.98 (0.93, 1.02) | 13 | 0.318 | 0.339 |
| Drug-related TEAE | 2 | 334 | 252 | 0.99 (0.88, 1.12) | 0 | 0.758 | 0.910 |
| SAE | 2 | 297 | 213 | 0.80 (0.33, 1.95) | 0 | 0.844 | 0.623 |
| Immunogenicity outcome | | | | | | | |
| ADA | 3 | 459 | 371 | 0.93 (0.85, 1.02) | 0 | 0.442 | 0.132 |
| Nabs | 3 | 411 | 322 | 0.93 (0.72, 1.19) | 0 | 0.648 | 0.546 |

Note: NA, not applicable; PASI, psoriasis area and severity index; PASI75, 75% improvement in PASI score; ACR20, American College of Rheumatology 20% Response Criteria; TEAE, Treatment Emergent Adverse Event; SAE, Serious Adverse Event; ADA, Anti-Drug antibody; Nabs, Neutralizing antibodies.

**eTable S9.** **Weighted mean price per unit of Adalimumab, Infliximab, Tocilizumab, and their biosimilars between 2015 and 2024.**

| Year | Infliximab ($/mg) | | | Tocilizumab ($/mg) | | | Adalimumab ($/mg) | | |
| --- | --- | --- | --- | --- | --- | --- | --- | --- | --- |
|  | Biosimilar | Reference | B/R (%) | Biosimilar | Reference | B/R (%) | Biosimilar | Reference | B/R (%) |
| 2024 | 1.79 | 2.83 | 63% | 1.15 | 1.46 | 79% | 3.70 | 4.54 | 82% |
| 2023 | 1.96 | 3.11 | 63% | 1.36 | 1.50 | 90% | 3.73 | 4.79 | 78% |
| 2022 | 1.88 | 2.97 | 63% | NA | 1.53 | NA | 3.69 | 5.00 | 74% |
| 2021 | 1.91 | 2.99 | 64% | NA | 1.79 | NA | 4.28 | 5.34 | 80% |
| 2020 | NA | 3.01 | NA | NA | 1.78 | NA | 4.31 | 7.62 | 57% |
| 2019 | NA | 8.43 | NA | NA | 1.63 | NA | 4.91 | 9.68 | 51% |
| 2018 | NA | 8.59 | NA | NA | 3.85 | NA | NA | 30.67 | NA |
| 2017 | NA | 8.74 | NA | NA | 3.91 | NA | NA | 31.14 | NA |
| 2016 | NA | 10.08 | NA | NA | 3.98 | NA | NA | 32.26 | NA |
| 2015 | NA | 9.41 | NA | NA | 4.03 | NA | NA | 32.48 | NA |

Note: NA, not available; B, biosimilars; R, references.

**eTable S10. Weighted average monthly treatment costs of Adalimumab, Infliximab, Tocilizumab, and their biosimilars for patients with Rheumatoid Arthritis in 2024.**

| Infliximab vs Infliximab biosimilars | | | |
| --- | --- | --- | --- |
| Indications | Biosimilar (1.79$/mg) | Reference (2.83$/mg) | Save money ($) |
| Rheumatoid arthritis | 3430 | 5423 | 1993 |
| Crohn's disease in Adults | 5717 | 9038 | 3322 |
| Fistula Crohn's disease | 5717 | 9038 | 3322 |
| Ankylosing spondylitis | 5717 | 9038 | 3322 |
| Plaque psoriasis | 5717 | 9038 | 3322 |
| Ulcerative colitis | 5717 | 9038 | 3322 |
| Adalimumab vs. Adalimumab biosimilars | | | |
| Indications | Biosimilar (3.70$/mg) | Reference (4.54$/mg) | Save money ($) |
| Rheumatoid arthritis | 3848 | 4722 | 874 |
| Ankylosing spondylitis | 3848 | 4722 | 874 |
| Plaque psoriasis | 3848 | 4722 | 874 |
| Tocilizumab vs. Tocilizumab biosimilars | | | |
| Indications | Biosimilar (1.20$/mg) | Reference (1.50$/mg) | Save money ($) |
| Rheumatoid arthritis | 8736 | 10920 | 2184 |

| Infliximab vs Infliximab biosimilars | | | |
| --- | --- | --- | --- |
| Indications | Biosimilar (1.79$/mg) | Reference (2.83$/mg) | Save money ($) |
| Rheumatoid arthritis | 3430 | 5423 | 1993 |
| Crohn's disease in Adults | 5717 | 9038 | 3322 |
| Fistula Crohn's disease | 5717 | 9038 | 3322 |
| Ankylosing spondylitis | 5717 | 9038 | 3322 |
| Plaque psoriasis | 5717 | 9038 | 3322 |
| Ulcerative colitis | 5717 | 9038 | 3322 |
| Adalimumab versus Adalimumab biosimilars | | | |
| Indications | Biosimilar (3.70$/mg) | Reference (4.54$/mg) | Save money ($) |
| Rheumatoid arthritis | 3848 | 4722 | 874 |
| Ankylosing spondylitis | 3848 | 4722 | 874 |
| Plaque psoriasis | 3848 | 4722 | 874 |
| Tocilizumab versus Tocilizumab biosimilars | | | |
| Indications | Biosimilar (1.20$/mg) | Reference (1.50$/mg) | Save money ($) |
| Rheumatoid arthritis | 8736 | 10920 | 2184 |

**eTable S11. Uptake rates of Adalimumab, Infliximab, Tocilizumab, and their biosimilars between 2015 and 2024.**

| Time frames of market entry | Date of market entry | Biosimilar, mg | Reference, mg | Overall^a^, mg | B/O^b^ (%) |
| --- | --- | --- | --- | --- | --- |
| Adalimumab biosimilar vs. Adalimumab | | | | | |
| M_1-3_ | 2020_Q1_ | 22400 | 338200 | 360600 | 6.21% |
| M_4-6_ | 2020_Q2_ | 327920 | 726240 | 1054160 | 31.11% |
| M_7-9_ | 2020_Q3_ | 769525 | 1316815 | 2086340 | 36.88% |
| M_10-12_ | 2020_Q4_ | 1072975 | 1477943 | 2550918 | 42.06% |
| M_13-15_ | 2021_Q1_ | 1564242 | 1836417 | 3400659 | 46.00% |
| M_16-18_ | 2021_Q2_ | 2256643 | 1970996 | 4227639 | 53.38% |
| M_19-21_ | 2021_Q3_ | 3026120 | 2072332 | 5098453 | 59.35% |
| M_22-24_ | 2021_Q4_ | 2786109 | 2027165 | 4813275 | 57.88% |
| M_25-27_ | 2022_Q1_ | 2940550 | 2215761 | 5156311 | 57.03% |
| M_28-30_ | 2022_Q2_ | 3151405 | 2196521 | 5347925 | 58.93% |
| M_31-33_ | 2022_Q3_ | 3824750 | 2616676 | 6441426 | 59.38% |
| M_34-36_ | 2022_Q4_ | 3510852 | 2107631 | 5618482 | 62.49% |
| M_37-39_ | 2023_Q1_ | 4235172 | 2203100 | 6438272 | 65.78% |
| M_40-42_ | 2023_Q2_ | 5070795 | 2274241 | 7345036 | 69.04% |
| M_43-45_ | 2023_Q3_ | 5379260 | 2272020 | 7651280 | 70.31% |
| M_46-48_ | 2023_Q4_ | 5077120 | 2006860 | 7083980 | 71.67% |
| M_49-51_ | 2024_Q1_ | 5685080 | 2196960 | 7882040 | 72.13% |
| M_52-54_ | 2024_Q2_ | 5925240 | 2295580 | 8220820 | 72.08% |
| Infliximab biosimilar vs. Infliximab | | | | | |
| M_1-3_ | 2021_Q4_ | 11685 | 7689513 | 7701198 | 0.15% |
| M_4-6_ | 2022_Q1_ | 80882 | 7861225 | 7942108 | 1.02% |
| M_7-9_ | 2022_Q2_ | 170671 | 6976569 | 7147240 | 2.39% |
| M_10-12_ | 2022_Q3_ | 455075 | 7884438 | 8339512 | 5.46% |
| M_13-15_ | 2022_Q4_ | 571162 | 6387319 | 6958481 | 8.21% |
| M_16-18_ | 2023_Q1_ | 1027015 | 7170718 | 8197733 | 12.53% |
| M_19-21_ | 2023_Q2_ | 1132524 | 6582232 | 7714756 | 14.68% |
| M_22-24_ | 2023_Q3_ | 1420200 | 7114000 | 8534200 | 16.64% |
| M_25-27_ | 2023_Q4_ | 1400900 | 7712800 | 9113700 | 15.37% |
| M_28-30_ | 2024_Q1_ | 1572800 | 8416400 | 9989200 | 15.75% |
| M_31-33_ | 2024_Q2_ | 1778300 | 8987200 | 10765500 | 16.52% |
| Tocilizumab biosimilar vs. Tocilizumab | | | | | |
| M_1-3_ | 2023_Q1_ | 592080 | 9783933 | 10376013 | 5.71% |
| M_4-6_ | 2023_Q2_ | 558902 | 5052295 | 5611197 | 9.96% |
| M_7-9_ | 2023_Q3_ | 1101520 | 5974640 | 7076160 | 15.57% |
| M_10-12_ | 2023_Q4_ | 6263040 | 5432160 | 11695200 | 53.55% |
| M_13-15_ | 2024_Q1_ | 7364240 | 6542880 | 13907120 | 52.95% |
| M_16-18_ | 2024_Q2_ | 7980080 | 6988800 | 14968880 | 53.31% |

Note: M, month. Q, quarter. The subscripts M and Q represent month and quarter respectively, since the biosimilar was marketed. a, Represents the sum of sale volumes of biosimilars and reference drugs. b, Represents the uptake of biosimilars.

**eTable S12. Savings and additional patient benefits under various biosimilar substitution scenarios.**

|  | Calculation indicator (unit) | Adalimumab | Infliximab | Tocilizumab |
| --- | --- | --- | --- | --- |
| Basic index | A=Sales revenue of originator in 2023 ($) | 39495290 | 76818782 | 40033051 |
|  | B=Sales volume of originator in 2023 (mg) | 8756220 | 27178849 | 26243027 |
|  | C=Sales revenue of biosimilar in 2023 ($) | 73213672 | 8838042 | 3631082 |
|  | D=Sales volume of biosimilar in 2023 (mg) | 19762347 | 4980639 | 8515541 |
|  | E=Annual treatment cost of originator in 2023 ($) | 4982 | 5423 | 10920 |
|  | F=Weighted annual treatment cost of biosimilar drugs in 2023 ($)* | 3430 | 3430 | 8736 |
| First scenario: biosimilar substitution in 2023 | |  |  |  |
| Savings in healthcare costs after substitution | Savings = D* (A/B - C/D) | 15925205 | 5239325 | 9359153 |
| Number of additional patients benefiting after substitution | Number of additional patients benefiting = Savings/F | 4643 | 1527 | 1071 |
| Second scenario: 100% of biosimilar substitution | |  |  |  |
| Savings in healthcare costs after simulated substitution | Savings = (B+D)*(A/B-C/D) | 22981280 | 33829796 | 38202005 |
| Number of additional patients benefiting after substitution | Number of additional patients benefiting = Savings/F | 6700 | 9863 | 4373 |

**eTable S13. Comparison of approved indications for IMIDs originators and biosimilars by the NMPA.**

| Types | Reference | Biosimilars | | | | | | |
| --- | --- | --- | --- | --- | --- | --- | --- | --- |
| Adalimumab and Adalimumab biosimilars | | | | | | | | |
| Indications | Humira  (Abbvie) | Geleli (Bio-Thera) [BAT1406] | Anjianning (Hisun) [HS016] | Sulixin (Innovent Bio) [IBI303] | Handayuan (Henlius Biotech) [HLX03] | Taibowei (CTTQ Pharma) [TQZ2301] | Junmaikang (Suzhou Union ) [UBP1211] | Anjiarun (Sinocelltech Group) [SCT630] |
| Rheumatoid arthritis | 2010/3/12 | 2019/11/13 | 2019/12/6 | 2020/9/2 | 2020/12/7 | 2022/1/18 | 2022/3/3 | 2023/6/12 |
| Ankylosing spondylitis | 2013/4/11 | 2019/11/13 | 2019/12/6 | 2020/9/2 | 2020/12/7 | 2022/1/18 | 2022/3/3 | 2023/6/12 |
| Plaque psoriasis | 2017/5/19 | 2019/11/13 | 2019/12/6 | 2020/9/2 | 2020/12/7 | 2022/1/18 | 2022/3/3 | 2023/6/12 |
| Juvenile idiopathic arthritis | 2019/11/8 | 2021/3/22 | 2021/7/30 | 2020/11/23 | 2024/2/29 | 2022/5/31 | 2022/11/21 | 2023/6/12 |
| Plaque psoriasis in children | 2020/3/27 | 2021/2/8 | 2021/3/5 | 2020/12/23 | 2024/2/29 | 2022/1/18 | 2022/11/21 | 2023/6/12 |
| Crohn's disease | 2020/1/13 | 2020/7/22 | 2020/5/6 | 2022/5/31 | 2024/2/29 | 2022/06/08 | 2022/11/21 | 2023/6/12 |
| Uveitis | 2020/3/27 | 2020/8/7 | 2021/7/30 | 2020/12/23 | 2021/4/8 | 2022/07/18 | 2022/11/21 | 2023/6/12 |
| Crohn's disease in children | 2021/4/23 | 2021-09-13 | 2021/7/30 | 2022/5/31 | 2024/2/29 | 2022/6/8 | 2022/11/21 | 2023/6/12 |
| Infliximab and Infliximab biosimilars | | | | | | | | |
| Indications | Remicade (Janssen) | Leiting (Taizhou Mabpharm Taike) [CMAB008] | Jiayoujian (Genor Bio) [GB242] | Anbaite (Hisun) [HS626] |  |  |  |  |
| Crohn's disease | 2006/5/3 | 2021/7/14 | 2022/2/28 | 2021/9/24 |  |  |  |  |
| Ankylosing spondylitis | 2006/5/3 | 2021/7/14 | 2022/2/28 | 2021/9/24 |  |  |  |  |
| Fistula Crohn's disease | 2006/5/3 | 2021/7/14 | 2022/2/28 | 2021/9/28 |  |  |  |  |
| Rheumatoid arthritis | 2006/5/3 | 2021/7/14 | 2022/2/28 | 2021/9/24 |  |  |  |  |
| Plaque psoriasis | 2013/8/4 | 2021/7/14 | 2022/2/28 | 2021/9/24 |  |  |  |  |
| Ulcerative colitis | 2018/12/24 | 2021/7/14 | 2022/2/28 | 2021/9/24 |  |  |  |  |
| Tocilizumab and Tocilizumab biosimilars | | | | | | | | |
| Indications | Actemra (Roche) | Anweitai (Livzon) [LZM008] | Shiweili (Bio-Thera) [BAT1806] |  |  |  |  |  |
| Rheumatoid arthritis | 2013/3/26 | 2023/1/19 | 2023/1/17 |  |  |  |  |  |
| Juvenile idiopathic arthritis | 2016/11/1 | 2023/1/19 | 2023/1/17 |  |  |  |  |  |

Note: IMIDs, Immune-Mediated Inflammatory Diseases; NMPA, National Medical Products Administration.

**eTable S14. Reimbursable IMIDs indications for Adalimumab, Infliximab, and Tocilizumab biosimilars and reference drugs in China’s National Reimbursement Drug List (As of January 1, 2024).**

| Originator | Approved indications | Reimbursable indications |
| --- | --- | --- |
| Adalimumab | Rheumatoid arthritis  Ankylosing spondylitis  Plaque psoriasis  Juvenile idiopathic arthritis  Plaque psoriasis in children  Crohn's disease  Uveitis  Crohn's disease in children | Rheumatoid arthritis  Ankylosing spondylitis  Plaque psoriasis  Juvenile idiopathic arthritis  Plaque psoriasis in children  Crohn's disease  Uveitis  Crohn's disease in children |
| Infliximab | Crohn's disease  Ankylosing spondylitis  Fistula Crohn's disease  Rheumatoid arthritis  Ulcerative colitis | Crohn's disease  Ankylosing spondylitis  Fistula Crohn's disease  Rheumatoid arthritis  Ulcerative colitis |
| Tocilizumab | Rheumatoid arthritis  Juvenile idiopathic arthritis | Restricted to patients diagnosed definitively with rheumatoid arthritis and treated with conventional DMARDs for 3-6 months, with a decrease in disease activity of less than 50%  Second-line treatment of systemic juvenile idiopathic arthritis |

Note: IMIDs, Immune-Mediated Inflammatory Diseases; DMARDs, Disease-modifying antirheumatic drugs.

Figure

**eFigure S1. Flowchart of included studies for the systematic review.**

Note: IMIDs, Immune-Mediated Inflammatory Diseases.


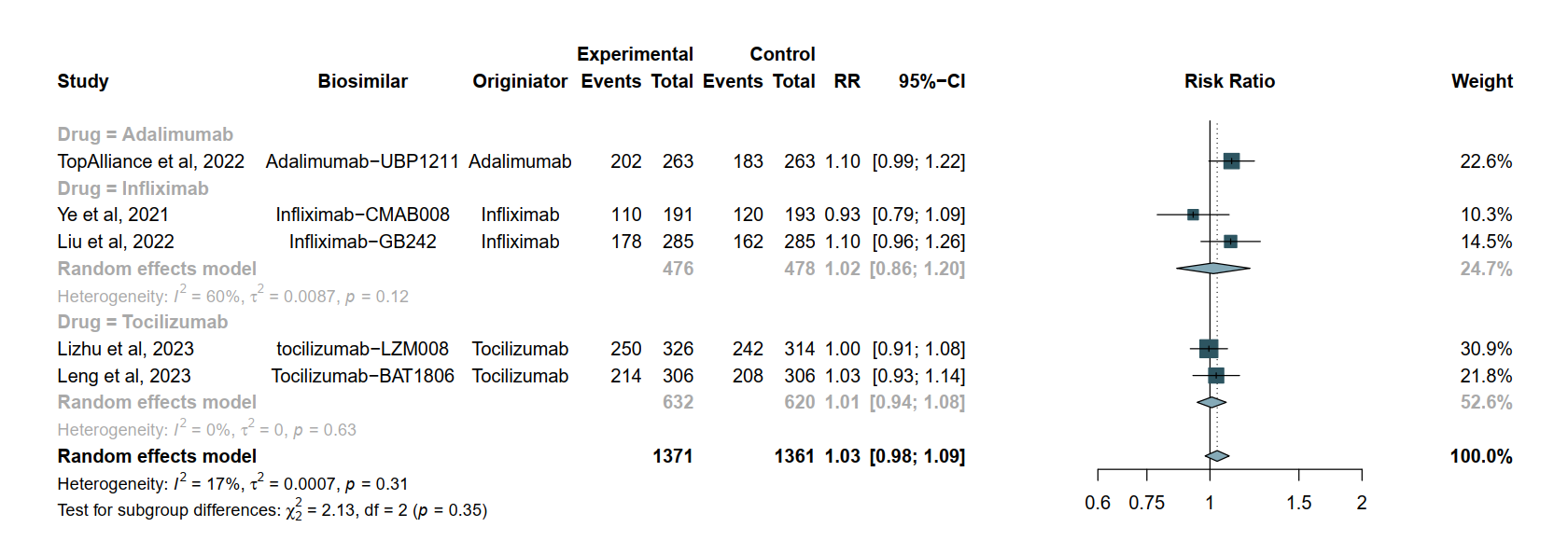


**eFigure S2. Forest plots for the american college of rheumatology 20% response criteria (ACR20) for primary endpoints at 24 weeks after treatments with biosimilars and their reference drug.**


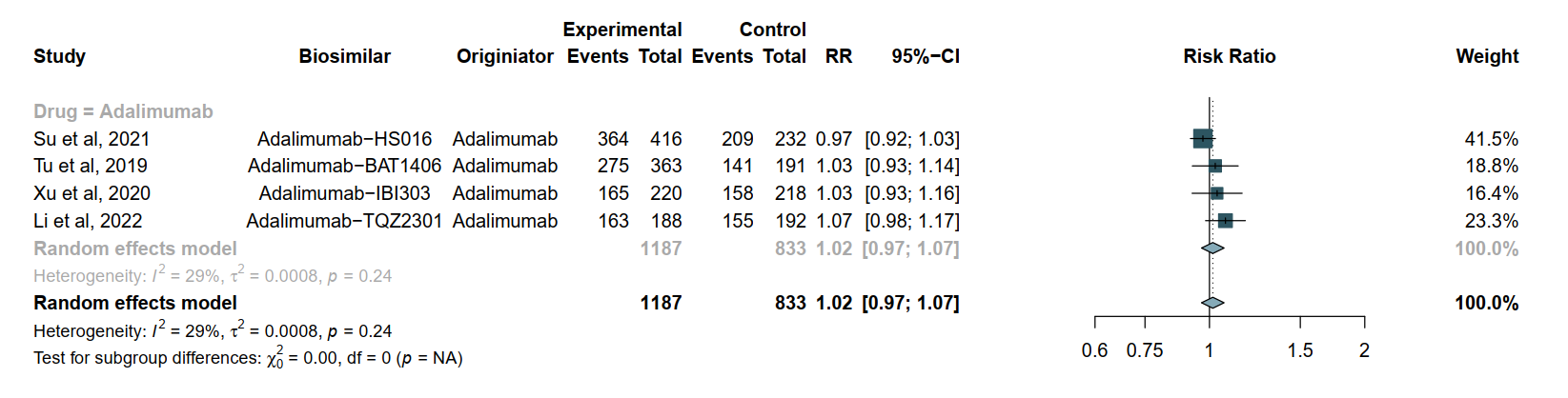


**eFigure S3. Forest plots for the assessment of spondylo arthritis international society (ASAS) for primary endpoints at 24 weeks after treatments with biosimilars and their reference drug.**


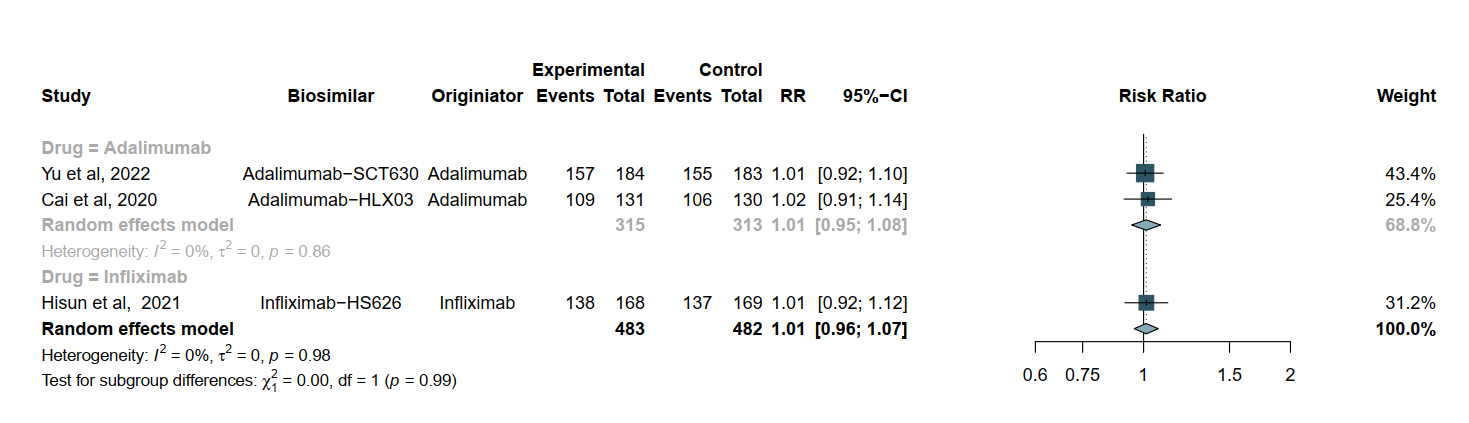


**eFigure S4. Forest plots of psoriasis area and severity index（PASI）for primary endpoints after treatments with biosimilars and their reference drug.**


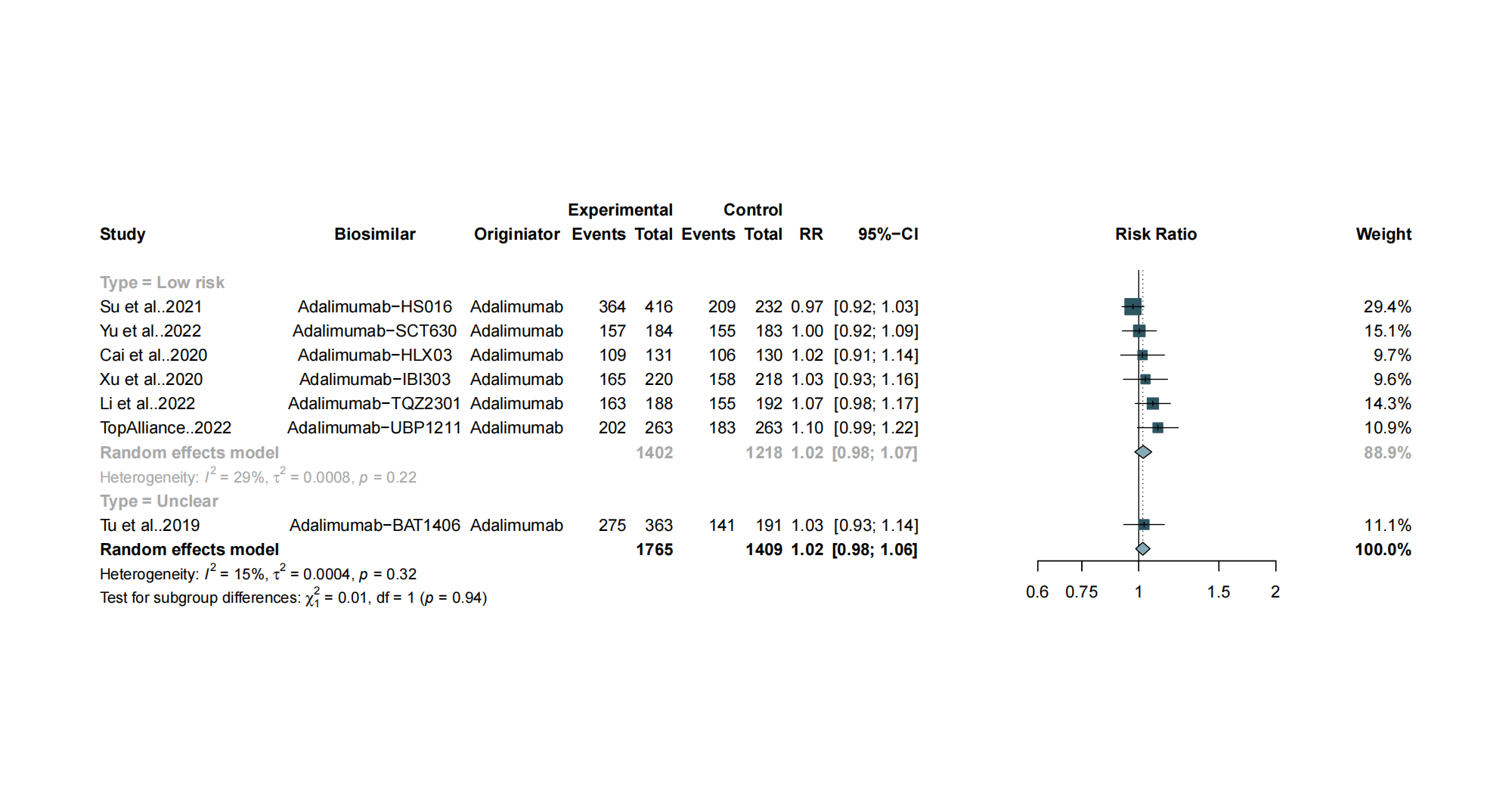


**eFigure S5. Subgroup analysis of adalimumab biosimilar based on risk of bias assessment results.**


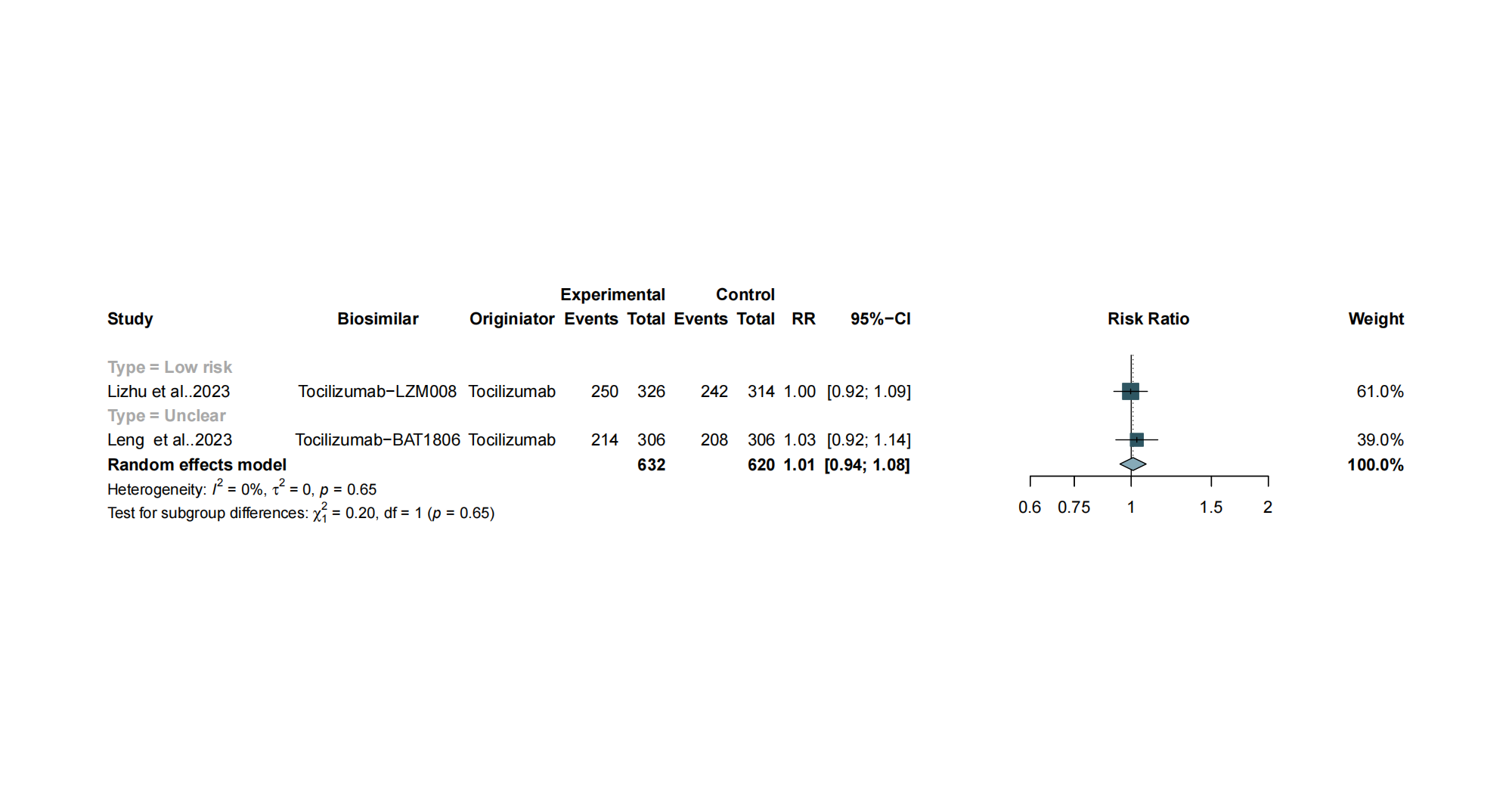


**eFigure S6. Subgroup analysis of torizumab biosimilar based on risk of bias assessment results.**

**Reference**

1. The database of listed drugs. China National Medical Products Administration. https://www.cde.org.cn/main/xxgk/listpage/b40868b5e21c038a6aa8b4319d21b07d. Accessed January 1, 2024.

2. Ascef BO, Almeida MO, Medeiros-Ribeiro AC, Oliveira de Andrade DC, Oliveira Junior HA, de Soarez PC. Therapeutic Equivalence of Biosimilar and Reference Biologic Drugs in Rheumatoid Arthritis: A Systematic Review and Meta-analysis. *JAMA Netw Open.* 2023;6(5):e2315872.

3. Luo X, Du X, Li Z, et al. Clinical Benefit, Price, and Uptake for Cancer Biosimilars vs Reference Drugs in China: A Systematic Review and Meta-Analysis. *JAMA Netw Open.* 2023;6(10):e2337348.

4. Meng Q, Mills A, Wang L, Han Q. What can we learn from China’s health system reform? *BMJ.* 2019;365:l2349.

5. State Administration of Foreign Exchange. Renminbi Exchange Rate Intermediate Price. https://www.safe.gov.cn/safe/rmbhlzjj/index.html. Accessed January 1, 2024.

6. Yu C, Zhang F, Ding Y, et al. A randomized, double-blind phase III study to demonstrate the clinical similarity of biosimilar SCT630 to reference adalimumab in Chinese patients with moderate to severe plaque psoriasis. *Int Immunopharmacol.* 2022;112:109248.

7. Li J, Xue Z, Wu Z, et al. Comparison of the efficacy and safety of the adalimumab biosimilar TQ-Z2301 and adalimumab for the treatment of Chinese patients with active ankylosing spondylitis: a multi-center, randomized, double-blind, phase III clinical trial. *Clin Rheumatol.* 2022;41(10):3005-3016.

8. Xu H, Li Z, Wu J, et al. IBI303, a biosimilar to adalimumab, for the treatment of patients with ankylosing spondylitis in China: a randomised, double-blind, phase 3 equivalence trial. *The Lancet Rheumatology.* 2019;1(1):e35-e43.

9. Su J, Li M, He L, et al. Comparison of the Efficacy and Safety of Adalimumab (Humira) and the Adalimumab Biosimilar Candidate (HS016) in Chinese Patients with Active Ankylosing Spondylitis: A Multicenter, Randomized, Double-Blind, Parallel, Phase III Clinical Trial. *BioDrugs.* 2020;34(3):381-393.

10. Cai L, Li L, Cheng H, et al. Efficacy and Safety of HLX03, an Adalimumab Biosimilar, in Patients with Moderate-to-Severe Plaque Psoriasis: A Randomized, Double-Blind, Phase III Study. *Adv Ther.* 2022;39(1):583-597.

11. Huang F, Sun F, Wan W, et al. Fri0414 Secukinumab Provides Rapid and Significant Improvement in the Signs and Symptoms of Ankylosing Spondylitis: Primary (16-Week) Results from a Phase 3 China-Centric Study, Measure 5. Spondyloarthritis – treatment; 2019.

12. China National Medical Products Administration.review reports of Adalimumab biosimilar. https://www.cde.org.cn/main/xxgk/postmarketpage?acceptidCODE=4e535af6c6b64d430b967bda35039e1f. Accessed January 1, 2024.

13. Liu Y, Liu S, Liu L, et al. Fine Comparison of the Efficacy and Safety Between GB242 and Infliximab in Patients with Rheumatoid Arthritis: A Phase III Study. *Rheumatol Ther.* 2022;9(1):175-189.

14. China National Medical Products Administration. Review reports of Infliximab biosimilar. https://www.cde.org.cn/main/xxgk/postmarketpage?acceptidCODE=24db4eca5de0823cad843b37efbafa08. Accessed January 1, 2024.

15. China National Medical Products Administration.review reports of Infliximab biosimilar. https://www.cde.org.cn/main/xxgk/postmarketpage?acceptidCODE=a3ab02b753af80706f30073b45a7d140. Accessed January 1, 2024.

16. Ye H, Liu S, Xu J, et al. Efficacy and Safety of CMAB008 Compared with Innovator Infliximab in Patients with Moderate-to-Severe Rheumatoid Arthritis Receiving Concomitant Methotrexate: A Randomized, Double-blind, Multi-center, Phase III Non-inferiority Study. *Rheumatol Ther.* 2023;10(3):757-773.

17. Meng C, Rajesh D, Jannat-Khah D, Bruce O, Jivanelli B, Bykerk V. Pos0286 Can Patients with Controlled Ra Receiving Any Class of Targeted Therapy with Methotrexate (Mtx) Sustain Disease Control after Tapering Mtx? A Systematic Review and Meta-Analysis. *Annals of the Rheumatic Diseases.* 2022;81(Suppl 1):387-388.

18. China National Medical Products Administration. Review reports of Tocilizumab biosimilar. https://www.cde.org.cn/main/xxgk/postmarketpage?acceptidCODE=7d426010254d5e4dceddabf06c0ce702. Accessed January 1, 2024.
